# Insights from the largest diverse ancestry sex-specific disease map for genetically predicted height

**DOI:** 10.1038/s41525-025-00464-w

**Published:** 2025-02-27

**Authors:** A. Papadopoulou, E. M. Litkowski, M. Graff, Z. Wang, R. A. J. Smit, G. Chittoor, I. Dinsmore, N. S. Josyula, M. Lin, J. Shortt, W. Zhu, S. L. Vedantam, L. Yengo, A. R. Wood, S. I. Berndt, I. A. Holm, F. D. Mentch, H. Hakonarson, K. Kiryluk, C. Weng, G. P. Jarvik, D. Crosslin, D. Carrell, I. J. Kullo, O. Dikilitas, M. G. Hayes, W. -Q. Wei, D. R. V. Edwards, T. L. Assimes, J. N. Hirschhorn, J. E. Below, C. R. Gignoux, A. E. Justice, R. J. F. Loos, Y. V. Sun, S. Raghavan, P. Deloukas, K. E. North, E. Marouli

**Affiliations:** 1 William Harvey Research Institute, Faculty of Medicine and Dentistry, Queen Mary University of London, London, UK; 2 VA Eastern Colorado Health Care System, Aurora, CO USA; 3 Division of Biomedical Informatics and Personalized Medicine, Department of Medicine, University of Colorado Anschutz Medical Campus, Aurora, CO USA; 4 Department of Epidemiology, University of North Carolina at Chapel Hill, Chapel Hill, NC USA; 5 The Charles Bronfman Institute for Personalized Medicine, Icahn School of Medicine at Mount Sinai, New York, NY USA; 6 The Mindich Child Health and Development Institute, Icahn School of Medicine at Mount Sinai, New York, NY USA; 7 Department of Clinical Epidemiology, Leiden University Medical Center Leiden, Leiden, NL The Netherlands; 8 The Genetics of Obesity and Related Metabolic Traits Program, Icahn School of Medicine at Mount Sinai, New York, NY USA; 9 Department of Population Health Sciences, Geisinger, Danville, PA USA; 10 Department of Genomic Health, Geisinger, Danville, PA USA; 11 Colorado Center for Personalized Medicine, Department of Biomedical Informatics, University of Colorado Anschutz Medical Campus, Aurora, USA; 12 Vanderbilt Genetics Institute, Vanderbilt University Medical Center, Nashville, TN USA; 13 Program in Medical and Population Genetics, Broad Institute of Harvard and MIT, Cambridge, MA USA; 14 Division of Endocrinology and Center for Basic and Translational Obesity Research, Boston Children’s Hospital, Boston, MA USA; 15 Institute for Molecular Bioscience, The University of Queensland, Brisbane, Australia; 16 Department of Biomedical Science, Centre of Membrane Interactions and Dynamics, University of Sheffield, Western Bank, Sheffield, UK; 17 Division of Cancer Epidemiology and Genetics, National Cancer Institute, NIH, Bethesda, MD USA; 18 Division of Genetics and Genomics and Manton Center for Orphan Diseases Research, Boston Children’s Hospital, Department of Pediatrics, Harvard Medical School, Boston, MA USA; 19 The Center for Applied Genomics, Children’s Hospital of Philadelphia, Philadelphia, PA USA; 20 Department of Medicine, Division of Nephrology, Vagelos College of Physicians & Surgeons, Columbia University, New York, NY USA; 21 Department of Biomedical Informatics, Vagelos College of Physicians & Surgeons, Columbia University, New York, NY USA; 22 Department of Medicine (Medical Genetics) and Genome Sciences, University of Washington Medical Center, Seattle, WA USA; 23 Division of Biomedical Informatics and Genomics, John W. Deming Department of Medicine, Tulane University, School of Medicine, New Orleans, LA USA; 24 Kaiser Permanente Washington Health Research Institute, Seattle, WA USA; 25 Department of Cardiovascular Medicine and the Gonda Vascular Center, Mayo Clinic, Rochester, MN USA; 26 Division of Endocrinology, Metabolism, and Molecular Medicine, Feinberg School of Medicine, Northwestern University, Chicago, IL USA; 27 Department of Biomedical Informatics, Vanderbilt University Medical Center, Nashville, TN USA; 28 Division of Quantitative Sciences, Department of Obstetrics and Gynecology, Vanderbilt University Medical Center, Nashville, TN USA; 29 VA Palo Alto Health Care System, Palo Alto, CA USA; 30 Program in Medical and Population Genetics, Broad Institute, Boston, MA USA; 31 Departments of Genetics and Pediatrics Harvard Medical School, Boston, MA USA; 32 Novo Nordisk Foundation Center for Basic Metabolic Research, Faculty of Health and Medical Sciences, University of Copenhagen, Copenhagen, Denmark; 33 Atlanta VA Health Care System, Decatur, GA USA; 34 Department of Epidemiology, Emory University Rollins School of Public Health, Atlanta, GA USA; 35 Digital Environment Research Institute, Queen Mary University of London, London, UK

**Keywords:** Genetics, Diseases

## Abstract

We performed ancestry and sex specific Phenome Wide Association Studies (PheWAS) to explore disease related outcomes associated with genetically predicted height. This is the largest PheWAS on genetically predicted height involving up to 840,000 individuals of diverse ancestry. We explored European, African, East Asian ancestries and Hispanic population groups. Increased genetically predicted height is associated with hyperpotassemia and autism in the male cross-ancestry analysis. We report male-only European ancestry associations with anxiety disorders, post-traumatic stress and substance addiction and disorders. We identify a signal with benign neoplasm of other parts of digestive system in females. We report associations with a series of disorders, several with no prior evidence of association with height, involving mental disorders and the endocrine system. Our study suggests that increased genetically predicted height is associated with higher prevalence of many clinically relevant traits which has important implications for epidemiological and clinical disease surveillance and risk stratification.

## Introduction

Adult height is an easily measured anthropometric trait that is complex and highly heritable^1,2^. Several factors contribute to adult height including both genetic and environmental, such as nutrition, socio-economic status, and physical activity^3–7^.

Several observational studies have been performed to better understand the association between height and disease. In individuals of European ancestry, increased height has been associated with a reduced risk of several circulatory diseases, including coronary artery disease (CAD), aortic valve stenosis (AS), heart failure (HF), hypertension and stroke^6^. In addition to these observational studies, increased genetically predicted height has been associated with decreased risk of hypertension, diaphragmatic hernia, and gastro-esophageal reflux disease (GERD)^6^. A recent study in the Million Veteran Program (MVP) used a polygenic score based on 3290 height-associated Single Nucleotide Polymorphisms (SNPs)^2^ to show that increased genetically predicted height is associated with an increased risk of atrial fibrillation (AF) and decreased risk of CAD, hypertension, hyperlipidemia. They also reported potential novel associations with peripheral neuropathy and infections of the skin and bones, both in European and African ancestries individuals^8^. Furthermore, increased genetically predicted height has been associated with longer PR interval and QRS duration^9^, venous thromboembolism^6,10^, AF, intervertebral disc disorder, hip fracture, vasculitis, breast cancer^6,11^ and colorectal cancer^6,12^ in European ancestry.

The Genetics of Anthropometric Traits - (GIANT) - consortium has performed increasingly larger meta-analyses of genome-wide association studies (GWAS) of height over the years^2,13,14^. In the present study we are using a multi-ancestry polygenic score (PGS) for height in six study populations of diverse ancestries to explore the association with a comprehensive set of health-related outcomes. The PGS for height was constructed using genetic variants taken from the most recent GIANT GWAS for adult height, excluding data from 23andMe^14^. We employed a Phenome-Wide Association Analyses (PheWAS) approach; a hypothesis-free analysis, with no prior assumptions, to detect phenotypes associated with the height PGS^15,16^, followed by meta-analysis (meta-PheWAS) of the individual PheWAS in each study population both within and across-ancestry groupings, to potentially identify new diseases associated with genetically predicted height. Sex-stratified cross-ancestry analyses were also considered.

## Results

**Fig. 1:**
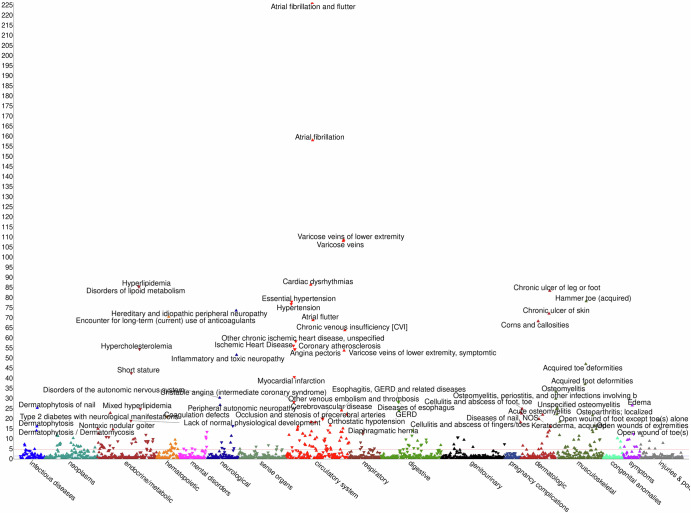
Phenome Wide Association Study (PheWAS). Manhattan plot showing the significant phecodes per category for the sex-combined cross-ancestry PheWAS meta-analysis of European (EUR), African (AFR), East Asian (EAS) ancestries and Hispanic (HIS) population groups.

We performed a PheWAS in each cohort using the PGS of height as exposure and tested its association with disease outcomes available in each of them (Methods). The sex-combined cross-ancestry meta-PheWAS in up to 839,872 participants, interrogating 1768 traits (available in at least 2 cohorts), yielded 254 significant associations below Bonferroni threshold (*p*-value = 2.83E-05) (Table 1, Fig. 1). All phecode categories harbored multiple significant associations with the tested height PGS; circulatory system (62), congenital anomalies (4), dermatologic (17), digestive (18), endocrine/metabolic (35), genitourinary (8), hematopoietic (11), infectious diseases (6), injuries & poisonings (10), mental disorders (7), musculoskeletal (29), neoplasms (16), neurological (11), respiratory (7), sense organs (6) and symptoms (7) (Supplementary Data 6).

**Table 1:** Sample size and number of traits in the cross-ancestry meta-PheWAS

Population	Sample	Number of traits	Significant traits	Bonferroni threshold
Sex-combined	839,872	1768	254	2.83 × 10^–5^
Males	471,395	1582	173	3.16 × 10^–5^
Females	267,576	1499	56	3.34 × 10^–5^

The traits that displayed the strongest associations with height PGS are shown in Table 2. The results from the PheWAS performed in each cohort, along with the full results from the meta-PheWAS, are presented in Supplementary Data 7– Supplementary Data 14 and Supplementary Data 6, respectively.

**Table 2:** Top 20 significant hits from the sex-combined cross-ancestry PheWAS meta-analysis of EUR, AFR, EAS, HIS

phecode	Description	Category	OR	Lower	Upper	*P*-value	pval het
427.2	Atrial fibrillation and flutter	Circulatory system	1.16	1.15	1.17	1.08 × 10^–226^	4.50 × 10^–4^
427.21	Atrial fibrillation	Circulatory system	1.15	1.14	1.17	1.24 × 10^–158^	1.27 × 10^–3^
454.1	Varicose veins of lower extremity	Circulatory system	1.16	1.15	1.18	2.87 × 10^–109^	4.17 × 10^–2^
454	Varicose veins	Circulatory system	1.15	1.14	1.17	1.23 × 10^–108^	5.85 × 10^–2^
427	Cardiac dysrhythmias	Circulatory system	1.07	1.06	1.07	5.68 × 10^–87^	3.63 × 10^–10^
272.1	Hyperlipidemia	Endocrine/metabolic	0.94	0.94	0.95	4.04 × 10^–86^	1.34 × 10^–4^
272	Disorders of lipoid metabolism	Endocrine/metabolic	0.94	0.94	0.95	7.13 × 10^–86^	1.28 × 10^–4^
707.2	Chronic ulcer of leg or foot	Dermatologic	1.19	1.17	1.21	6.29 × 10^–84^	7.27 × 10^–3^
735.21	Hammer toe (acquired)	Musculoskeletal	1.18	1.16	1.20	9.23 × 10^–79^	3.12 × 10^–3^
401.1	Essential hypertension	Circulatory system	0.95	0.94	0.95	1.51 × 10^–78^	1.29 × 10^–3^
401	Hypertension	Circulatory system	0.95	0.94	0.95	2.20 × 10^–77^	1.31 × 10^–3^
356	Hereditary and idiopathic peripheral neuropathy	Neurological	1.15	1.13	1.16	3.71 × 10^–74^	1.21 × 10^–1^
707	Chronic ulcer of skin	Dermatologic	1.14	1.12	1.16	1.19 × 10^–72^	1.05 × 10^–5^
286.2	Encounter for long-term (current) use of anticoagulants	Hematopoietic	1.13	1.12	1.15	4.40 × 10^–71^	8.36 × 10^–4^
427.22	Atrial flutter	Circulatory system	1.17	1.15	1.19	1.96 × 10^–69^	1.18 × 10^–1^
700	Corns and callosities	Dermatologic	1.15	1.13	1.17	7.51 × 10^–69^	2.42 × 10^–5^
456	Chronic venous insufficiency [CVI]	Circulatory system	1.16	1.14	1.18	2.04 × 10^–64^	6.65 × 10^–2^
411.8	Other chronic ischemic heart disease, unspecified	Circulatory system	0.93	0.92	0.94	6.38 × 10^–59^	2.80 × 10^–1^
411	Ischemic Heart Disease	Circulatory system	0.95	0.94	0.95	1.03 × 10^–56^	6.53 × 10^–3^
411.4	Coronary atherosclerosis	Circulatory system	0.94	0.94	0.95	5.14 × 10^–55^	2.07 × 10^–3^

From the cross-ancestry meta-PheWAS analysis, six traits exhibited evidence of heterogeneity (defined as when the *p*-value of the Cochran’s heterogeneity test is below Bonferroni threshold) as shown in Table 3, Supplementary Figs. 1–7. For example, in *cardiac dysrhythmias (427)* the signal indicated strong evidence for association in European ancestry (*p*-value = 1.29 × 10^–91^) but not in the other ancestral groups (Supplementary Data 4). We also observed evidence of heterogeneity of effects across cohorts for Cardiac dysrhythmias (Supplementary Fig. 3). Another notable example of heterogeneity was for *Chronic ulcer of skin (707)* (Supplementary Fig. 5).

**Table 3:** Heterogeneous traits in the cross-ancestry meta-PheWAS analysis

phecode	Description	Category	OR	Lower	Upper	*p*-value	pval het	I^2^
351	Other peripheral nerve disorders	Neurological	0.97	0.96	0.97	6.52 × 10^–17^	1.15 × 10^–17^	88.3%
427	Cardiac dysrhythmias	Circulatory system	1.07	1.06	1.07	5.68 × 10^–87^	3.63 × 10^–10^	81.9%
700	Corns and callosities	Dermatologic	1.15	1.13	1.17	7.51 × 10^–69^	2.42 × 10^–5^	73.2%
707	Chronic ulcer of skin	Dermatologic	1.14	1.12	1.16	1.19 × 10^–72^	1.05 × 10^–5^	73.3%
735	Acquired foot deformities	Musculoskeletal	1.06	1.05	1.07	1.11 × 10^–37^	2.62 × 10^–10^	82.1%
735.2	Acquired toe deformities	Musculoskeletal	1.10	1.09	1.11	1.32 × 10^–47^	1.93 × 10^–5^	72.4%

Cross-ancestry analyses revealed 30 additional signals that were not present in the European ancestry meta-analyses (Supplementary Data 5, Supplementary Fig. 8).

We further performed sex-specific meta-PheWAS analyses in the UKB, MVP, BioVU and BioMe cohorts (males: Supplementary Data 21, 23, 25, 27*and females:* Supplementary Data 32, 34, 36, 38*)*. The meta-PheWAS analysis for males in up to 471,395 participants, interrogated 1582 traits (available in at least two cohorts) and yielded 173 statistically significant trait associations below the Bonferroni threshold (*p*-value = 3.16 × 10^–5^) (Table 1) (Supplementary Data 19). The identified categories included the circulatory system (50), congenital anomalies (4), dermatologic (14), digestive (10), endocrine/metabolic (22), genitourinary (6), hematopoietic (6), infectious diseases (5), injuries & poisonings (6), mental disorders (8), musculoskeletal (21), neoplasms (5), neurological (7), respiratory (3), sense organs (3) and symptoms (3). In total, 10 traits, spanning across different categories, were significant only in the male cross-ancestry meta-PheWAS and not in the sex-combined cross-ancestry meta-PheWAS (Supplementary Data 42). For example, increased genetically predicted height was associated with decreased risk of *Hyperpotassemia (276.13)* (OR = 0.95, 95% CI [0.93, 0.97], *p*-value = 1.23 × 10^–6^, het *p*-value = 9.46 × 10^–1^) in males but showed a null association in females (Supplementary Data 42).

**Fig. 2:**
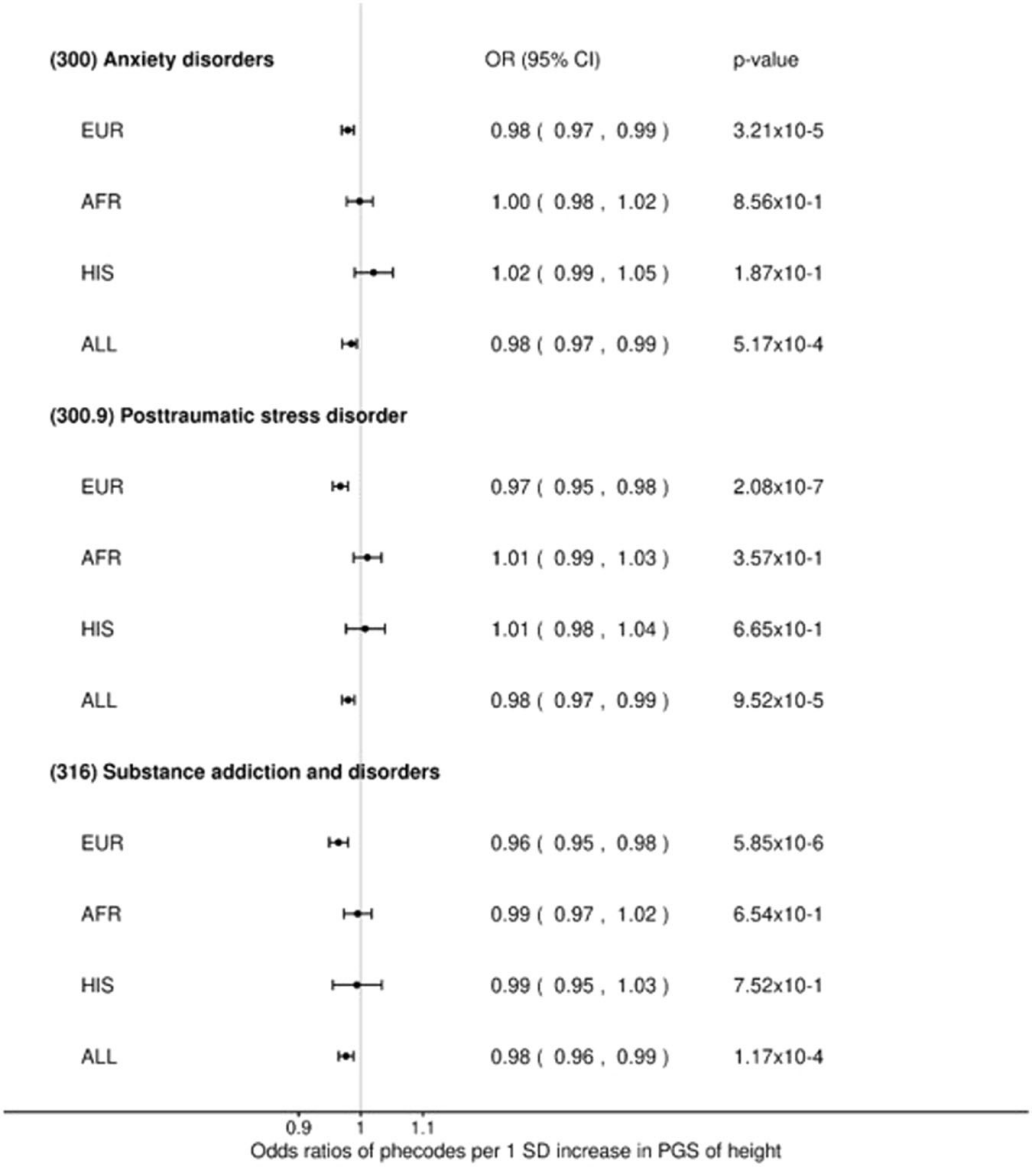
Estimates per ancestry in the male meta-analysis of Phenome Wide Association Studies (meta-PheWAS), for signals from the mental disorders category that were identified as significant only in the European males meta PheWAS. SD standard deviation of the mean, PGS polygenic score.

Looking at the ancestry level, 10 traits were significant (*p*-value < 3.16 × 10^–5^) only in European ancestry males but not in the male cross-ancestry analysis; 3 of them from the mental disorders category, with increased height PGS having a decreased risk of: *Anxiety disorders (300)*, *Posttraumatic stress disorder* (*300.9)* and *Substance addiction and disorders (316)* (Supplementary Data 16) (Fig. 2). In the other populations, *Drusen (degenerative) of retina (362.27)* and *Fracture of lower limb (800)* were significantly associated with increased height PGS in the male African (decreased risk) and Hispanic (increased risk) populations, respectively, but not in the cross-ancestry analyses (Supplementary Data 17 and 18).

The meta-PheWAS analysis in up to 267,576 female individuals, interrogated 1499 traits (available in at least two cohorts) and yielded 56 significant associations below Bonferroni threshold (*p*-value = 3.34 × 10^–5^) (Table 1) (Supplementary Data 30). The identified categories included the circulatory system (23), dermatologic (2), digestive (7), endocrine/metabolic (6), genitourinary (1), hematopoietic (1), musculoskeletal (5), neoplasms (8) and neurological (3). Only 1 association identified as significant in the females meta-PheWAS and was not observed in the sex-combined meta-PheWAS; *Benign neoplasm of other parts of digestive system (211)* (OR = 0.95, 95% CI [0.92, 0.97], *p*-value = 1.53 × 10^–5^, het *p*-value = 5.48 × 10^–1^) (Supplementary Data 41). Seven associations were significant in European ancestry but not in the cross-ancestry analysis, mainly from musculoskeletal and infectious diseases categories, such as *Osteoporosis (743.1)* and *Dermatophytosis / Dermatomycosis (110)*, respectively (Supplementary Data 29).

Comparing males to females, the meta-PheWAS yielded 126 significant associations only in males, primarily from the circulatory system, endocrine/metabolic and musculoskeletal categories (Supplementary Data 44). Ninety-three percent of the traits had concordant effect sizes and were larger for males. On the other hand, comparing females with males, the meta-PheWAS yielded 13 significant associations in females only, with the digestive and neoplasms categories including the most traits (Supplementary Data 43). Ninety-two percent of the traits were concordant in direction, and the effect sizes in females were larger. Examining the heterogeneity between males and females in the cross-ancestry meta-PheWAS, 7 associations were identified; 4 of them from the musculoskeletal category, such as *Acquired foot deformities (735)* (Supplementary Data 40).

We performed a meta-PheWAS analysis excluding UKB in the cross-ancestry sex-combined meta-PheWAS (Supplementary Data 46) and the sex-specific ones for males and females (Supplementary Data 50 and 54). For the sex-combined (Supplementary Fig. 9, Supplementary Data 46) and the males (Supplementary Fig. 10, Supplementary Data 50) meta-PheWAS the estimates are concordant as presented in the plots. In the cross-ancestry female meta-PheWAS (Supplementary Fig. 11, Supplementary Data 54) three traits were identified as discordant: Benign neoplasm of other parts of digestive system (211), Other disorders of circulatory system (459), Gastritis and duodenitis (535).

Replication analyses were performed in an independent sample of the Colorado Biobank. Comparing the European ancestry meta-PheWAS with the European PheWAS in Colorado biobank we observe that the ORs are concordant in their majority; Colorado biobank has larger error bars due to the smaller sample size than the meta-PheWAS analysis (Supplementary Figs. 12–14). Colorado biobank also provided PheWAS results using both weighted and unweighted PGS (Supplementary Figs. 15–17).

## Discussion

We performed a large ancestrally diverse meta-PheWAS for height in six cohorts including up to 840,000 individuals. Of the 1768 disease traits that were in common across cohorts and were meta-analysed, we identified 254 significant PGS-trait associations (*p*-value = 2.83 × 10^–5^). The largest number and most precise phenotypic associations were observed for the circulatory system, endocrine/metabolic and musculoskeletal categories.

From the circulatory system category, increased genetically predicted height was associated with an increased risk of *Chronic venous insufficiency (CVI) (456)* (OR = 1.16 95% CI [1.14, 1.18], *p*-value = 2.04 × 10^–64^) (Supplementary Data 6), with no evidence of heterogeneity across cohorts (het *p*-value = 6.65 × 10^–2^). These findings were concordant with a recent study in MVP which reported an association between increased genetically predicted height and increased risk of CVI in European American (EA) (OR = 1.366, *p*-value = 1.6 × 10^–35^) and in African American (AA) individuals (OR = 1.469, *p*-value = 3.1 × 10^–4^)^8^. The effect was similar in both males and females in our analyses. Failure of the femoral vein valves may lead to CVI, with severe consequences. However, for the valves to be replaced, the femoral vein diameter (FVD) must be known. A recent study by Keiler et al.^17^ reported that height was positively correlated with FVD; this correlation was attenuated when the sample was stratified by sex. In addition, failure of the venous valve can lead to varicose veins^17^. In our study, increased genetically predicted height was associated with increased risk of *Varicose veins (VV) (454)* (OR = 1.15, 95% CI [1.14, 1.17], *p*-value = 1.23 × 10^–108^) (Supplementary Data 6), with no evidence of heterogeneity across cohorts (het *p*-value = 5.85 × 10^–2^), again a finding in agreement with the MVP PheWAS^8^. Moreover, Mendelian Randomisation (MR) studies in European ancestry have supported a causal association between genetically predicted height and VV^18,19^.

Within the circulatory system category, the strongest association was for *Atrial fibrillation and flutter (AF) (427.2)* (OR = 1.16, 95% CI [1.15, 1.17], *p*-value = 1.08 × 10^–226^) (Supplementary Data 6), with no evidence of heterogeneity across cohorts (het *p*-value = 4.50 × 10^–4^), and similar effect sizes in the sex-stratified meta-PheWAS. The aforementioned MVP study similarly reported an increased risk of AF in EA (OR = 1.381, *p*-value = 5.70 × 10^–84^) and in AA (OR = 1.352, *p*-value = 3.3 × 10^–4^)^8^. Significant causal associations from MR analysis have been reported in two previous studies^6,20^.

Our study confirmed that increased genetically predicted height is inversely associated with cardiovascular diseases^21–23^. Increased genetically predicted height was associated with decreased risk of *hypertension (401)* (OR = 0.950, 95% CI [0.944, 0.955], *p*-value = 2.20 × 10^–77^) (Supplementary Data 6), with no evidence of heterogeneity across cohorts (het *p*-value = 1.31 × 10^–3^), and with similar effect sizes in males and females. This finding is in accordance with previous studies, although our effect sizes were slightly attenuated, possible due to a lack of coding “hypertension” using ICD codes^8,24^. According to World Health Organisation (WHO) “hypertension is diagnosed if, when it is measured on two different days, the systolic blood pressure readings on both days is ≥140 mmHg and/or the diastolic blood pressure readings on both days is ≥90 mmHg”^25^. A study in the Finnish population examining blood pressure found that shorter participants had higher SBP than taller ones, and this could be partially the reason for observing inverse association between height and cardiovascular disease^21^. A study in the USA reported that height was inversely associated with DBP in older males and females, in contrast to SBP that was positively associated^22^. A recent systematic review concluded that there was a potentially inverse association of stature and BP^26^. An MR analysis conducted in European ancestry individuals showed that an increase in adult height was causally associated with a lower risk of coronary heart disease, with one potential mechanism including BP^27^.

Epidemiological and genetic studies suggest that increased height is associated with decreased risk of CAD^6,23,28^. In a meta-analysis of European ancestry participants, genetically predicted increased height was associated with decreased risk of CAD (OR = 0.88, 95% CI [0.82, 0.95], *p*-value < 1.00 × 10^–3^)^28^. Similar findings were reported in several MR studies^6,23^. CAD is a broad category including diseases such as ischemic heart disease, myocardial infarction and coronary atherosclerosis. For instance, *Ischemic heart disease (411)* (OR = 0.948, 95%CI [0.942, 0.954], *p*-value = 1.03 × 10^–56^, het *p*-value = 6.53 × 10^–3^), and *Myocardial infarction (MI) (411.2)* (OR = 0.93, 95%CI [0.92, 0.94], *p*-value = 3.54 × 10^–41^, het *p*-value = 1.32 × 10^–1^) (Supplementary Data 6) were identified as significant among the cardiovascular diseases and with similar effect at the sex-stratified meta-PheWAS; all these have been confirmed in previous studies^8,29^.

In the endocrine/metabolic category, several health-related outcomes were identified. Our study identified decreased risk of *Hyperlipidemia (272.1)* (OR = 0.942, 95% CI [0.936, 0.947], *p*-value = 4.04 × 10^–86^, het *p*-value = 1.34 × 10^–4^) and *Hypercholesterolemia (272.11)* (OR = 0.946, 95% CI [0.939, 0.953], *p*-value = 5.67 × 10^–55^, het *p*-value = 4.17 × 10^–2^) (Supplementary Data 6), with similar effect at the sex-stratified meta-PheWAS. These findings have also been reported by MVP^8^, and in a Korean population^30,31^. Our meta-analysis confirmed the well-established association between 1 SD increase in genetically predicted height and decreased risk of *Type 2 diabetes (T2D) (250.2)* (OR = 0.98, 95% CI [0.97, 0.99], *p*-value = 2.27 × 10^–11^, het *p*-value = 9.11 × 10^–3^) (Supplementary Data 6)^32,33^. In addition, we observed an association between increased genetically predicted height and the increased risk of *Hypothyroidism (244)* (OR = 1.022, 95% CI [1.014, 1.031], *p*-value = 8.58 × 10^–8^, het *p*-value = 3.30 × 10^–1^) (Supplementary Data 6). This is an interesting insight towards the known epidemiological links between hypothalamic-pituitary-thyroid (HPT) axis dysregulation and stature^34^.

Several health outcomes from the musculoskeletal category were associated with genetically predicted height. *Acquired foot deformities (735)* (OR = 1.06, 95% CI [1.05, 1.07], *p*-value = 1.11 × 10^–37^) were associated with higher genetically predicted height, with strong evidence of heterogeneity across cohorts (het *p*-value = 2.62 × 10^–10^) (Supplementary Data 6). In the present study, EA descent individuals presented the strongest signal in MVP, followed by eMERGE and in AA only in MVP (Supplementary Fig. 6). We found this association in males only, which is supported by a previous study reporting foot deformities to be significantly more prevalent in male veterans versus male non-veterans in USA^35^. In contrast, *Osteoarthritis; localized (740.1)* (OR = 1.033, 95% CI [1.026, 1.039], *p*-value = 3.13 × 10^–22^, het *p*-value = 4.88 × 10^–2^) (Supplementary Data 6) was found to have a similar effect in both males and females. This finding is supported by the MVP PheWAS^8^ and is widely supported in the epidemiological literature, that taller individuals have an increased risk of knee osteoarthritis, that remained significant for both sexes, after adjusting for confounders^36^. A recent meta-analysis of GWAS studies for osteoarthritis, in Icelanders and European ancestry from UKB, found that a large proportion of osteoarthritis risk variants are associated with height^37^.

We identified several notable associations in the neoplasms category. There has been a significant body of literature studying the association between height and risk of breast cancer (BC) and the results are controversial. Several PheWAS and MR studies reported null associations between height PGS and BC^38,39^. In contrast, several studies, including ours, confirm the association of height and risk of BC. An observational study, using data from EPIC and the Women’s Health Initiative (WHI) in the USA, observed that for every 10 cm increase in height there was an 18% increased risk of ER + BC; null association was found for ER- BC^40^. Another observational study, analysing post-menopausal women from the Netherlands Cohort Study (1986-2006), observed that for every 5 cm increase in height there was a 7% increased risk of BC (95% CI: 1.01–1.13); an association that remained significant for the ER + BC but not for ER- BC^41^.

We observed an attenuated, non-significant association, between increased genetically predicted height and *Colorectal cancer (153)* (OR = 1.02, 95% CI [1.00, 1.04], *p*-value = 2.19 × 10^–2^, het *p*-value = 6.89 × 10^–1^) (Supplementary Data 6). This finding contrasts with the majority of PheWAS and MR studies that describe an association between increased adult height and increased risk of colorectal cancer^11,12,42^.

We identified a significant association between increased genetically predicted height and decreased risk of *Hyperpotassemia (276.13)* (OR = 0.95, 95% CI [0.93, 0.97], *p*-value = 1.23 × 10^–6^, het *p*-value = 9.46 × 10^–1^) (Supplementary Data 19) in males. Additionally, increased genetically predicted height was associated with 3 traits from the mental disorders category in the males meta-PheWAS: *Pervasive developmental disorders (313)* (OR = 1.06, 95% CI [1.03, 1.09], *p*-value = 6.11 × 10^–6^, het *p*-value = 5.25 × 10^–3^), *Attention deficit hyperactivity disorder (ADHD) (313.1)* (OR = 1.06, 95% CI [1.03, 1.09], *p*-value = 2.35 × 10^–5^, het *p*-value = 3.93 × 10^–1^) and *Autism (313.3)* (OR = 1.215, 95% CI [1.222, 1.316], *p*-value = 1.64 × 10^–6^, het *p*-value = 2.58 × 10^–1^) (Supplementary Data 19). Similarly, the traits were concordant in the sex-combined meta-PheWAS but showed null association in the female meta-PheWAS. Previous PheWAS provided suggestive support of these findings, with the exception of autism^8^. A study by Yackobovitch-Gavan et al.^43^ employing data from Israel Clalit Health Services, reported that drug treatment for ADHD was associated with greater decline of height z-score in boys than girls, with 66% of the participants being boys. Additionally two studies in the US, one for children^44^, and one for both children and adolescents^45^, confirmed a decline of height z-scores for patients using stimulants and it is confirmed by a study in Netherlands^46^. However, these studies have examined the case in which the participants are medicated. Nevertheless, there is evidence suggesting that there are more males diagnosed than females, which is in accordance with our results. Our results relating to autism are in accordance with the literature; a study in Spanish pre-school children showed that autism spectrum disorder (ASD) had increased height in contrast to children with typical development^47^. Additionally, in Australia, male babies with ASD were born smaller, but grew taller in comparison to children with typical development^48^. Therefore, for these disease traits, it seems that males drive the association.

In males, 10 phenotypes displayed significant associations with height PGS in European descent individuals only; 3 of them belong to the mental disorders category: *Anxiety disorders (300)* (OR = 0.98, 95% CI [0.97, 0.99], *p*-value = 3.21 × 10^–5^, het *p*-value = 1.40 × 10^–1^), *Posttraumatic stress disorder disorders* (*300.9)* (OR = 0.97, 95% CI [0.96, 0.98], *p*-value = 2.08 × 10^–7^, het *p*-value = 2.86 × 10^–1^) and *Substance addiction and disorders (316)* (OR = 0.96, 95% CI [0.95, 0.98], *p*-value = 5.85 × 10^–6^, het *p*-value = 9.10 × 10^–1^) (Supplementary Data 16).

Seven traits were identified as significant in the female meta-PheWAS for European descent individuals and not in the cross-ancestry analysis. Amongst them, increased height PGS was associated with decreased risk of *Osteoporosis (743.1)* (OR = 0.93, 95% CI [0.90, 0.96], *p*-value = 6.58 × 10^–6^, het *p*-value = 9.65 × 10^–1^) (Supplementary Data 29). Post-menopausal European ancestry females had an increased risk of osteoporotic fractures, in contrast to African and Asian ancestries^49,50^.

Our study had several important limitations. Although we used the recently published cross-ancestry GWAS from GIANT, the study populations were predominantly of European ancestry. Thus, we observed a poorer prediction performance of the height PGS in our study populations that were ancestrally diverse, diminishing the power in populations with substantial non-European admixture. It is also possible that some of the signals observed may be driven by differences in phenotype prevalence across cohorts. The differences in sample size by sex and ancestry complicate interpretation of differences across these strata. This limitation is not new for genetic studies but likely limits our inference on true sex and ancestry differences in the phenotype associations with genetically predicted height at phenome-wide significance. We included all available cohort data as a discovery meta-analysis to increase power. Trait associations with genetically predicted height may be particularly influenced by indirect genetic effects and assortative mating. A recent study showed that population estimates are larger than within-sibship meta-analysis GWAS estimates for height^51^. The authors presented strong evidence of polygenic adaptation on taller height in European ancestry individuals, suggesting that demographic effects, such as assortative mating, could vary between populations^51,52^. Additionally, previous work in the UK Biobank has reported an association between stature and socio-economic status in both sexes, therefore this could serve as a mediator of the reported associations rather than the actual direct effect of height^6^. Lastly, we did not consider obvious reasons for differences across studies, sex, and ancestry. Social factors have a powerful influence on many of the phenotype-genetically predicted height associations described herein. By including data from diverse populations in future investigation of the role of genetically predicted height across the phenome, future research might be able to address the limitations of this study. It might be possible to better understand the genetic and environmental factors that affect height by more broadly interpreting the results. The study’s power would be enhanced, and more precise results would be produced by expanding the sample size and providing more in-depth information on lifestyle factors. Finding associations between height and disease using data from different ancestries would improve the generalizability of our findings and offer a more thorough understanding of the genetic and environmental factors affecting height and disease risk. Additional approaches could include carrying out population-specific studies, which would enable the investigation of height-disease relationships in particular ethnic groups. This could be accomplished by enlisting volunteers from particular ethnic groups and gathering thorough data on disease outcomes, height, and other pertinent covariates like lifestyle variables. In the process of creating new treatments and preventative measures for a variety of diseases, this could assist in the identification of novel genetic variants and pathways.

## Methods

PheWAS is used to identify the effects of genetic variation already associated with a trait of interest across a larger array of phenotypes, using a hypothesis-free approach, with no prior assumptions^53^. We employed Bonferroni correction to determine statistical significance. Despite this, our large sample size facilitated the replication of known associations and even the discovery of new ones^54^.

To assess the associations of the PGS with hospital-record data, we used the PheWAS library^53^ implemented in R^55^. The package converts International Classification of Diseases (ICD) codes to ‘PheWAS codes’ or phecodes, which represent 1866 phenotypes in total formed from grouped ICD codes using the “Phecode Map 1.2 ICD-10-CM” (https://phewascatalog.org/phecodes_icd10cm). Each phenotype case is accompanied by accurate controls, meaning participants who have similar disease with the phenotype case are excluded. For instance, if the phenotype case under investigation is T2D, then participants who have T1D are excluded from the control group. This built-in exclusion feature, that prevents contamination of the controls, is essential to preserve statistical power to identify associations^53,56^. The phecodes are divided in 17 distinct categories: circulatory system, endocrine/metabolic, mental disorders, neurological, respiratory, infectious diseases, neoplasms, hematopoietic, sense organs, digestive, genitourinary, pregnancy complications, dermatologic, musculoskeletal, congenital anomalies, symptoms and injuries & poisonings^56^. Next, binary logistic regression models are employed to examine the association of the exposure, the PGS of height (independent variable), with the trait of interest with each phecode. As covariate adjustments in each study population, we used age, sex, genotype batch, to reduce model variability. Each study population (described in Supplementary Information) also adjusted for principal components for ancestry to control for confounding via population stratification (details per study on ancestry determination and exclusion in Supplementary Data 1A).

Details regarding compliance with all relevant ethical regulations including the Declaration of Helsinki can be found in the information and references for each participating cohort below. The PheWAS and meta-PheWAS summary statistics results that are discussed in the manuscript are included in the Supplementary Data 3–57.

### Polygenic score

We performed a conditional and joint analysis (GCTA-COJO) to select quasi-independent height-associated SNPs for the construction of the PGS^57,58^. A stepwise procedure was used for SNP selection and the joint effects of all selected SNPs were estimated after the model was optimized. The genetic variants are still genome-wide significant, independent and the variance explained by them is larger than considering only the leading SNP at each locus. This conditional analysis was performed in the recent cross-ancestry GWAS for adult height, excluding data from 23andMe^14^, using 50,000 unrelated and randomly sampled European participants of UKB as the LD reference panel. We performed analyses using *p*-value threshold *p* = 5 × 10^–9^ to declare a genome-wide significant hit. Also, SNPs with allele frequency differences larger than 0.2 as compared to a UKB reference panel, were excluded from the analysis along with SNPs having MAF ≤ 0.001. The GCTA-COJO analysis resulted in a list of 6797 SNPs. As covariate adjustments we used age, sex, genotype batch, to reduce model variability. We also adjusted for principal components for ancestry to control for confounding via population stratification (Supplementary Data 1B). The PGS of height was constructed as the unweighted sum of the height-increasing alleles within each study (Supplementary Data 1) and afterwards is was scaled (using scale function in R).

All herein reported ORs are per one standard deviation increase in PGS.

### Meta-analysis

Meta-analysis is a popular statistical technique used to increase the power to detect new effects by combining the information from independent studies. In addition, heterogeneity among the studies can be assessed, employing the beta estimates and standard errors from each study. For a small number of similar studies, the most common technique is the fixed-effect inverse variance weighted meta-analysis, which uses as a hypothesis that a common underlying effect exists for all studies^59,60^. We performed a meta-PheWAS, combined in a fixed-effect meta-analysis for UKB, MVP, BioVU, BioMe, MyCode and eMERGE cohorts, using the phecodes derived from the PheWAS in each cohort *(*Supplementary Data 3- Supplementary Data 14*)*. For the sex-specific analysis, we employed data from the UKB, MVP, BioVU and BioMe *(*Supplementary Data 15*–*38*)*. The examined ancestries were European, African, East Asian ancestries and Hispanic population groups, and the sample size per ancestry and per study are included in Table 4. For more details the reader is referred to Supplementary Data 1 and 2.

**Table 4:** Sample size per ancestry and per study

	EUR	AFR	EAS	HIS
**BioMe**	7993	6224		9007
**BioVU**	71,986	15,593		
**Emerge**	40,088			
**MVP**	221,927	57,974	5069	26,845
**MyCode**	46,922			
**UKB**	330,965	6682	1936	

The sample size and examined number of traits for the sex-combined and sex-specific cross-ancestry meta-PheWAS are detailed in Table 1, and for the specific ancestries in Supplementary Data 2. For the meta-analysis we employed the statistical software R 3.6.1 and the library *metafor*^61^.

### Replication

Replication analyses were performed in an independent sample of the Colorado Biobank. We also performed a replication PheWAS in the same biobank using a score weighted for the effects of the height-associated SNPs in the GWAS meta-analysis. Details are provided in the Supplementary Material.

#### UK Biobank (UKB)

The UKB is a prospective cohort of 502,504 participants, aged 40–69 years old, who were recruited between 2006 and 2010. The cohort includes information regarding a variety of phenotypes like blood measurements, clinical assessments, anthropometry, cognitive function, hearing, arterial stiffness, hand grip strength, spirometry, ECG, data on cancer and death registries, health and lifestyle medical conditions, operations, mental health, sociodemographic factors, lifestyle, family history, psychosocial factors and dietary intake, described in more detail elsewhere^62^. Hospital episode statistics (HES) is a database containing details of all admissions at NHS hospitals in UK, which has been linked to the UKB^63^.

#### Million Veteran Program (MVP)

The Department of Veterans Affairs (VA) created in 2011 a national cohort across USA: the MVP. This cohort was created as a representative, national and longitudinal study of Veterans for genomic and non-genomic research, employing responses to questionnaires, blood specimens and electronic health records (EHR). The blood specimens were collected for genotyping, and these were linked to the EHR, which coded the diagnosis in ICD9 and ICD10, up until September 2019. As expected, most of the participants are males, aged between 50 and 69 years old at recruitment. Regarding ethnicity, European Americans and African Americans are well represented; Hispanics and Asian descent participants are also included^64^.

The MVP study from Raghavan et al. ^8^ uses different sample than the one we are using in the current study.

#### BioVU

The Vanderbilt Institutional Review Board (IRB) approved the creation of Vanderbilt DNA databank, that collected DNA samples from 2007 until 2010. During the past years, the Vanderbilt University Medical Center has developed a comprehensive electronic medical record (EMR) system that covers all inpatient and outpatient data, including labs, drug ordering, and diagnostic imaging, including over 1.4 million records^65^. Regarding ethnicity, there is large concordance between race assignment and genetic ancestry for Europeans and African Americans, in contrast to lower concordance for Hispanics, East Asians and South Asians^66^.

#### BioMe

The Icahn School of Medicine at Mount Sinai’s Institutional Review Board approved in 2007 the construction of BioMe biobank. This EMR-linked biorepository enrolls participants non-selectively from the Mount Sinai Health System, which serves a diverse group of communities across the greater New York City area. At enrolment, participants provided informed consent to link their DNA and plasma sample to their EMR. This is further complemented by a questionnaire on demographic and lifestyle factors. At present, the cohorts comprise over 60,000 participants. 58% of the participants are females; participants were aged between 18 and 89+ years old at recruitment. Regarding ethnicity, European Americans, African Americans and Hispanics are well represented^67^.

#### Geisinger’s MyCode Community Health Initiative Study (MyCode)

The Geisinger Health System (GHS) includes a large percentage of stable participants from Pennsylvania, from more than 70 care facilities. In 2007 GHS initiated the MyCode Community Health Initiative (MyCode) to create a biobank of blood, serum, DNA samples along with genotype and exome sequence data. These data were linked to the EMR data for research purposes. By 2015, MyCode reported more than 90,000 participants and an ongoing monthly enrolment of around 2000, across the age spectrum (0 to >89 years old). Regarding ethnicity, more than 95% of the population are self-identified white or European American^68^.

#### Electronic Medical Records and Genomics (eMERGE) network

In 2007 the electronic MEdical Records and GEnomics (eMERGE) Network is a National Human Genome Research Institute (NHGRI) created to employ EHR for genomic research purposes. Today, eMERGE Network includes nine research groups across US, that they have connected the DNA samples to EHR. The majority of the studied participants have European ancestry, but also African, Asian and Hispanic descent participants are included in a smaller percent^69,70^.

#### Colorado Center for Personalized Medicine (CCPM Biobank)

The biobank at the Colorado Center for Personalized Medicine (CCPM Biobank) was jointly developed by the University of Colorado Anschutz Medical Campus and UCHealth to serve as a unique, dual-purpose research and clinical resource accelerating personalized medicine. As a resource comprising electronic health records (EHRs), genotype data, and other integrated data sources (e.g., geocoded data and survey data), the CCPM Biobank had more than 200,000 enrolled participants and 33,674 genotyped participants as of March 2022. The latter formed the freeze 2 research dataset. More details about the CCPM Biobank are described in Wiley et al. ^71^.

## Supplementary information

Supplementary Information

Supplementary Data 1

Supplementary Data 2

Supplementary Data 3

Supplementary Data 4

Supplementary Data 5

Supplementary Data 6

Supplementary Data 7

Supplementary Data 8

Supplementary Data 9

Supplementary Data 10

Supplementary Data 11

Supplementary Data 12

Supplementary Data 13

Supplementary Data 14

Supplementary Data 15

Supplementary Data 16

Supplementary Data 17

Supplementary Data 18

Supplementary Data 19

Supplementary Data 20

Supplementary Data 21

Supplementary Data 22

Supplementary Data 23

Supplementary Data 24

Supplementary Data 25

Supplementary Data 26

Supplementary Data 27

Supplementary Data 28

Supplementary Data 29

Supplementary Data 30

Supplementary Data 31

Supplementary Data 32

Supplementary Data 33

Supplementary Data 34

Supplementary Data 35

Supplementary Data 36

Supplementary Data 37

Supplementary Data 38

Supplementary Data 39

Supplementary Data 40

Supplementary Data 41

Supplementary Data 42

Supplementary Data 43

Supplementary Data 44

Supplementary Data 45

Supplementary Data 46

Supplementary Data 47

Supplementary Data 48

Supplementary Data 49

Supplementary Data 50

Supplementary Data 51

Supplementary Data 52

Supplementary Data 53

Supplementary Data 54

Supplementary Data 55

Supplementary Data 56

Supplementary Data 57

## Data Availability

Individual level data could be accessed upon request and approval from the respective biobanks. The summary statistics results that are discussed in the manuscript are included in the Supplementary Data.

## Code availability

All code for the analysis employed in this paper is publicly available (GCTA-COJO version: 1.93.0., PheWAS and metafor in R 3.6.1.).
