# Supplementary material for: Insights from the largest diverse ancestry sex-specific disease map for genetically predicted height
Source: NPJ Genom Med. 2025 Feb 27;10:14. doi: 10.1038/s41525-025-00464-w (PMC11868580; doi:10.1038/s41525-025-00464-w)
Supplement: Supplementary file 1 — Supplementary Information [file 41525_2025_464_MOESM1_ESM.pdf]

## Supplementary Figures

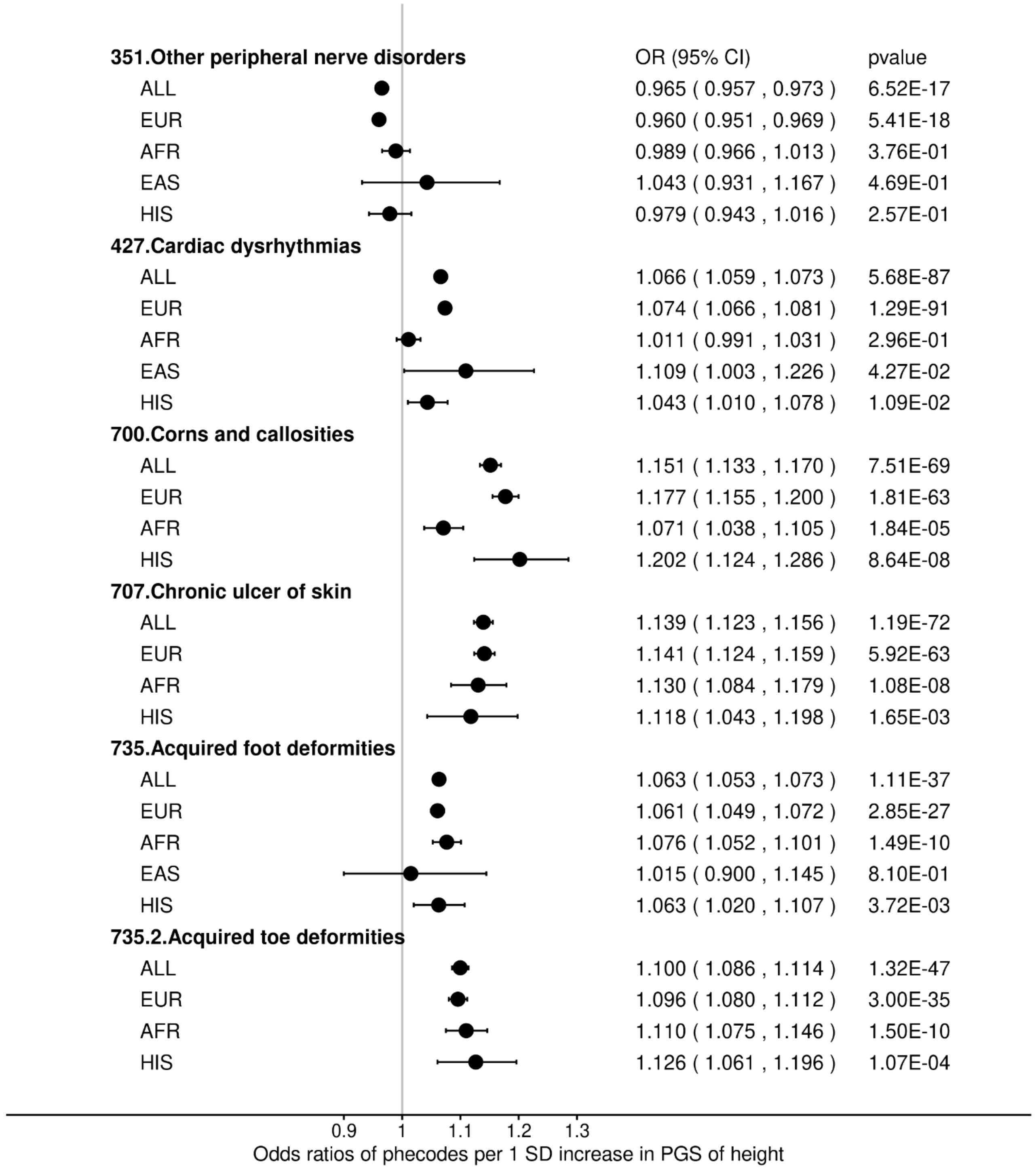


*Supplementary Figure 1: Traits with heterogeneity in sex-combined cross-sectional analysis and the corresponding estimates in the per ancestry sex-combined meta-analysis of Phenome Wide Association Studies (meta-PheWAS). SD; standard deviation of the mean, PGS; Polygenic Score.*

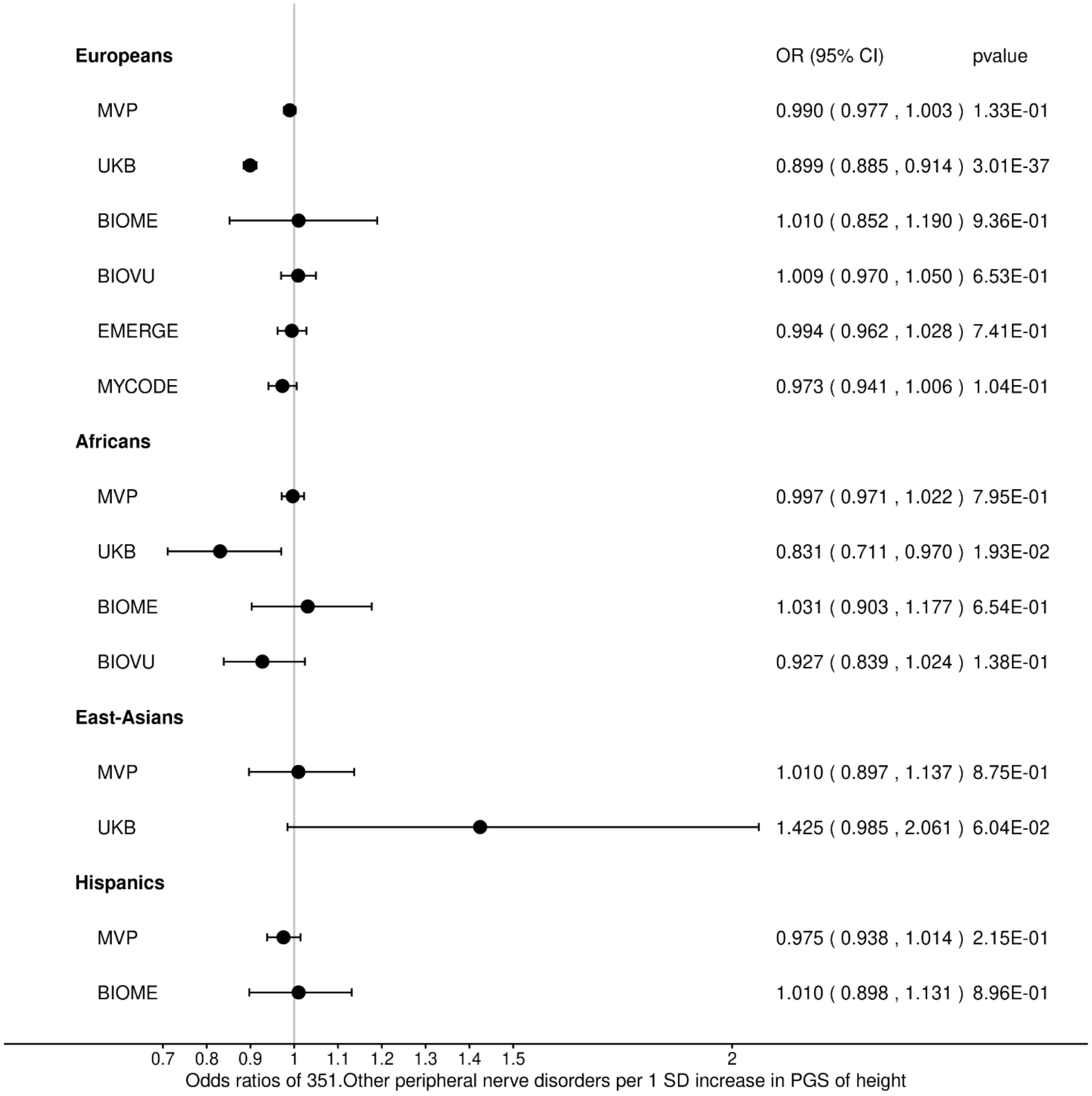


*Supplementary Figure 2: Odds Ratio (OR) estimates and 95% confidence intervals (CI) for (351) Other peripheral nerve disorders per ancestry and per cohort. SD; standard deviation of the mean, PGS; Polygenic Score.*

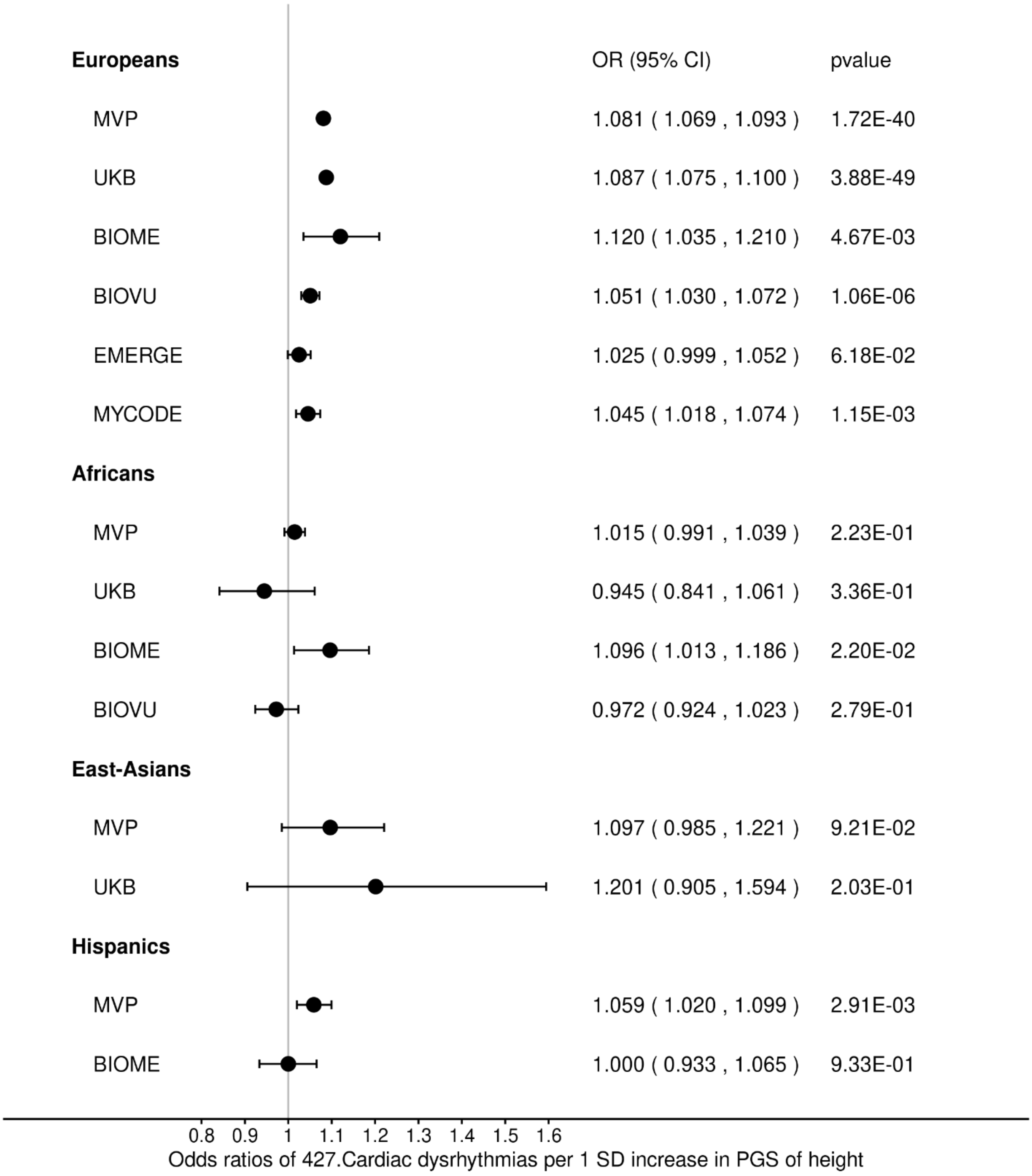


*Supplementary Figure 3: Odds Ratio (OR) estimates and 95% confidence intervals (CI) for (427) Cardiac dysrhythmias per ancestry and per cohort. SD; standard deviation of the mean, PGS; Polygenic Score.*

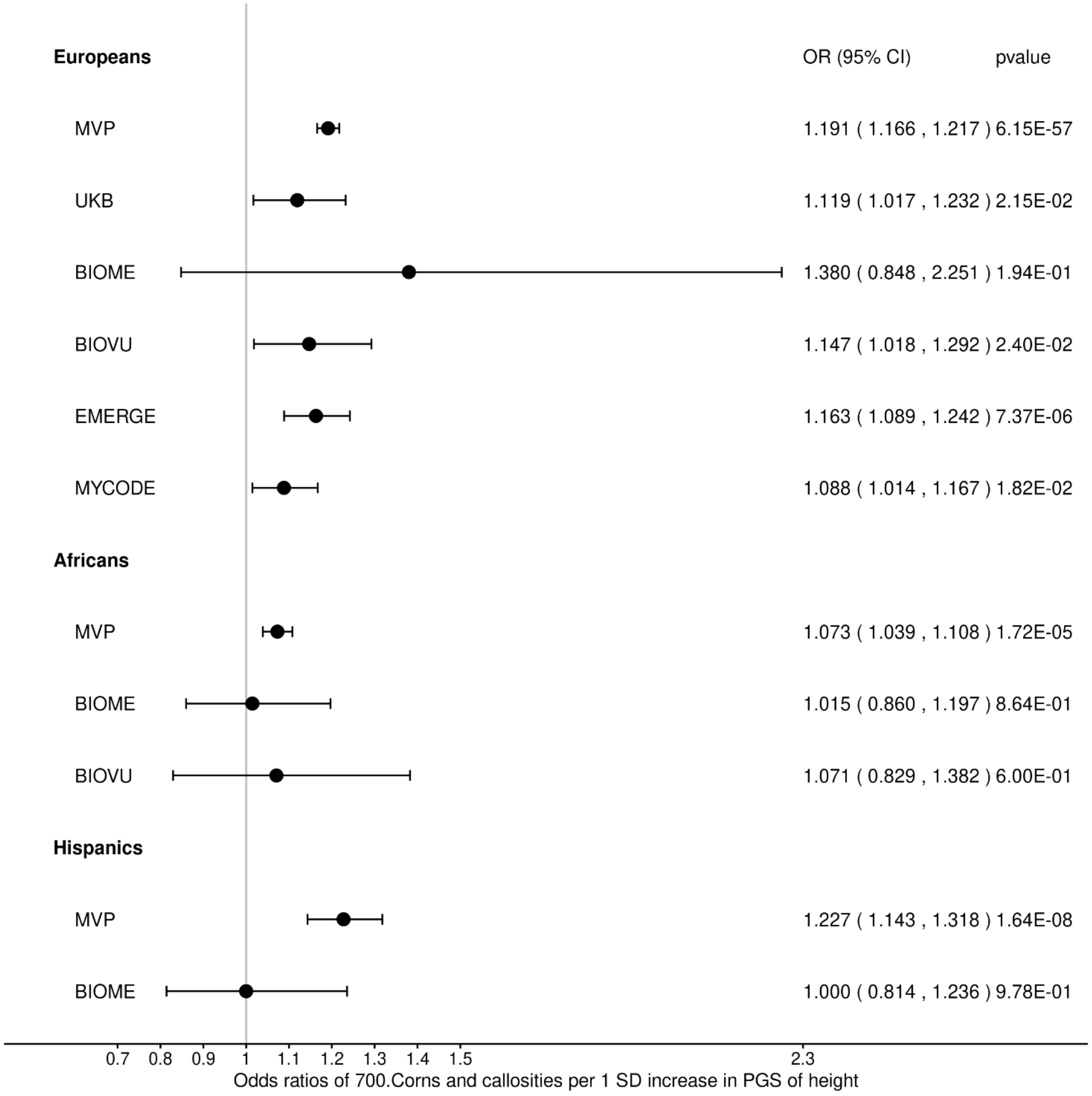


*Supplementary Figure 4: Odds Ratio (OR) estimates and 95% confidence intervals (CI) for (700) Corns and callosities per ancestry and per cohort. SD; standard deviation of the mean, PGS; Polygenic Score.*

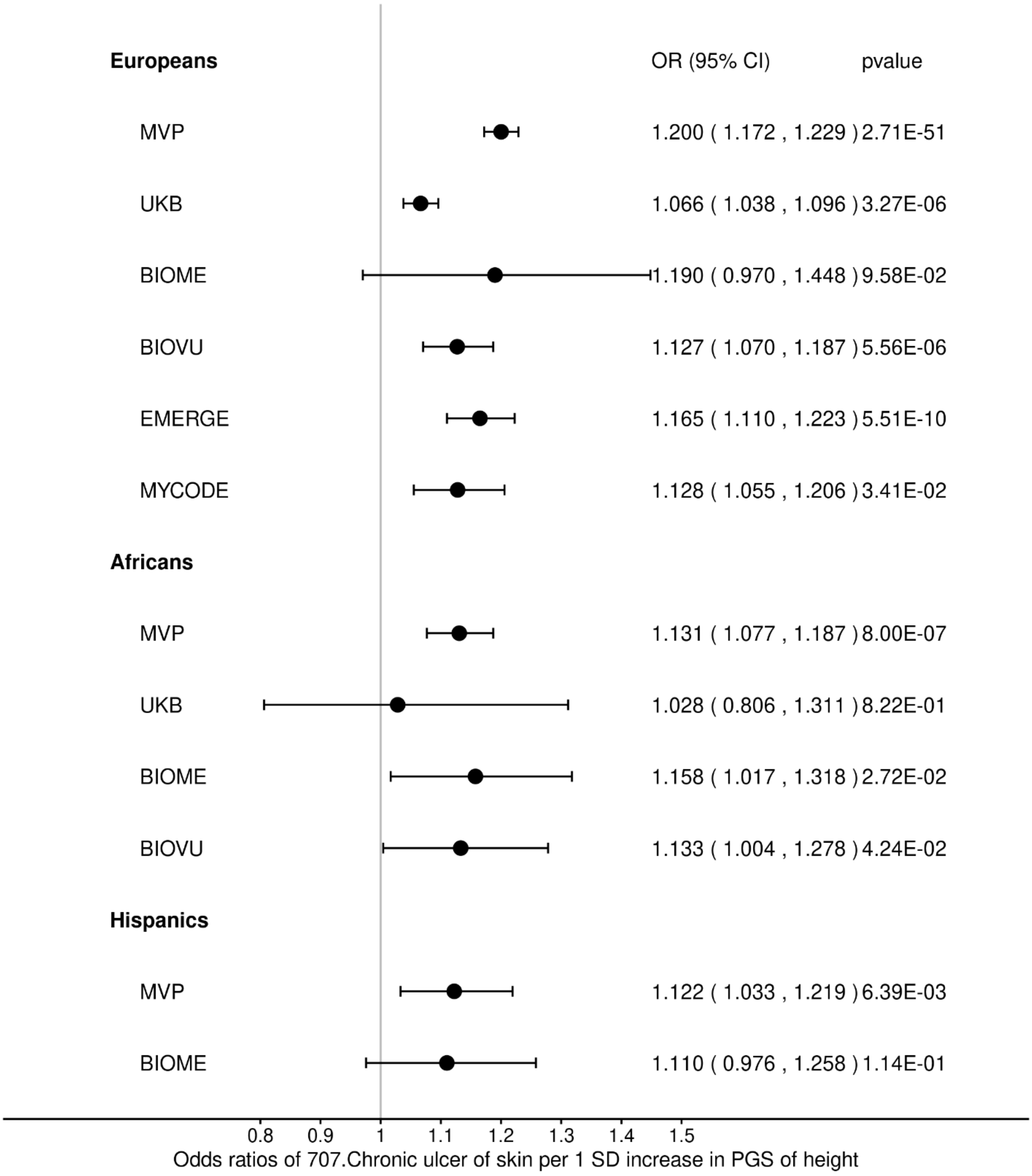


*Supplementary Figure 5: Odds Ratio (OR) estimates and 95% confidence intervals (CI) for (707) Chronic ulcer of skin per ancestry and per cohort. SD; standard deviation of the mean, PGS; Polygenic Score.*

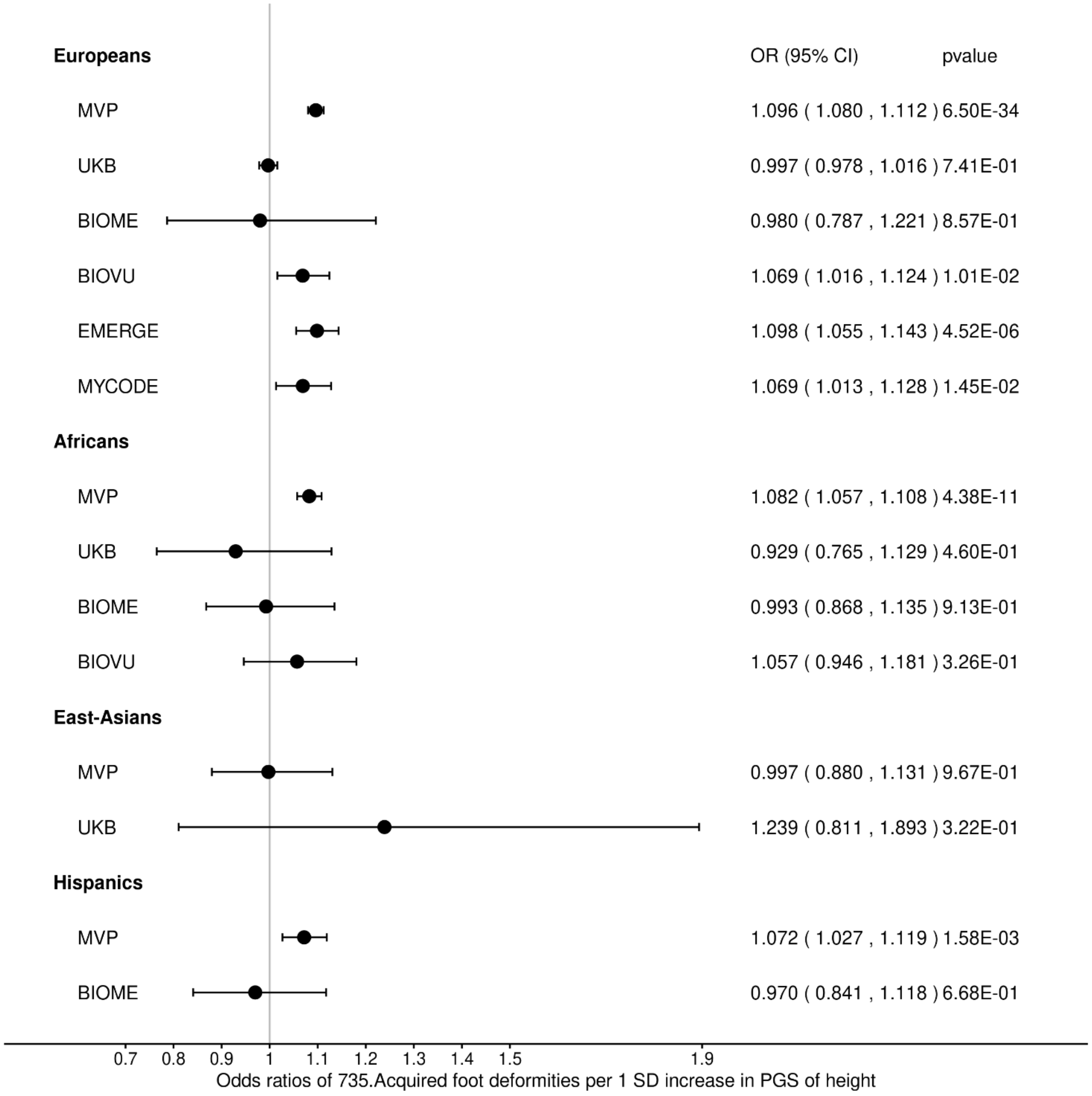


*Supplementary Figure 6: Odds Ratio (OR) estimates and 95% confidence intervals (CI) for (735) Acquired foot deformities per ancestry and per cohort. SD; standard deviation of the mean, PGS; Polygenic Score.*

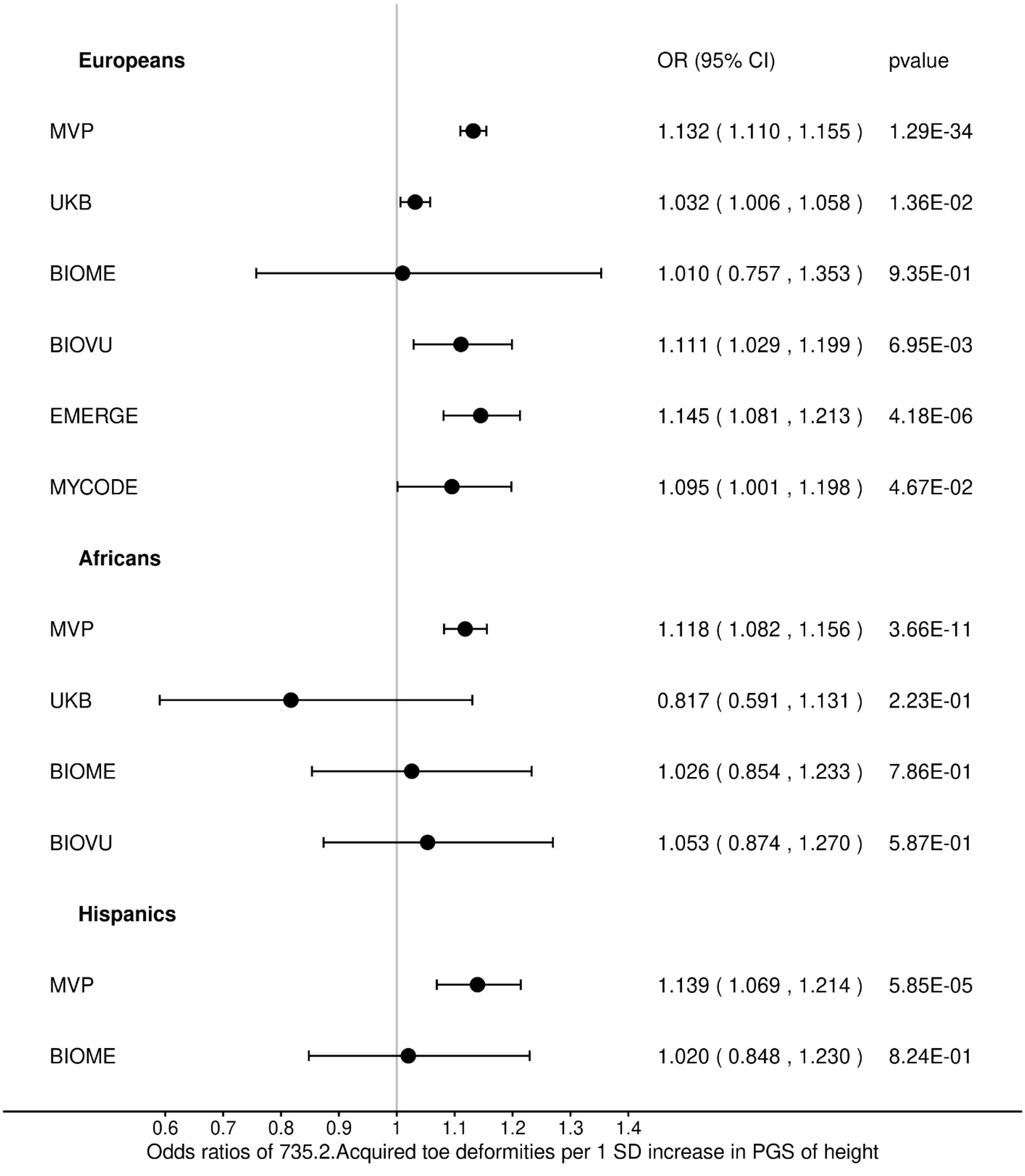


*Supplementary Figure 7: Odds Ratio (OR) estimates and 95% confidence intervals (CI) for (735.2) Acquired toe deformities per ancestry and per cohort. SD; standard deviation of the mean, PGS; Polygenic Score.*

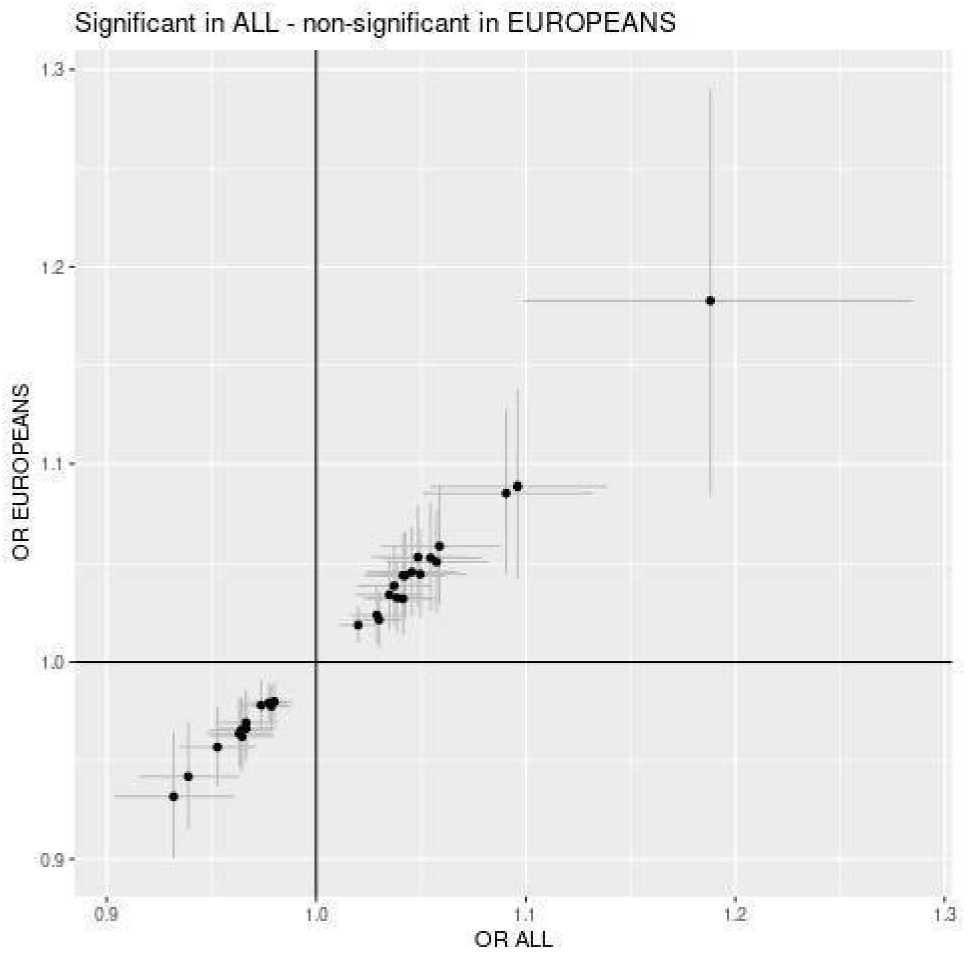


*Supplementary Figure 8: Signals that are below Bonferroni threshold for the sex-combined cross-ancestry meta-analysis of Phenome Wide Association Studies (meta-PheWAS) but not for the sex-combined European meta-PheWAS. The grey lines represent 95% confidence intervals.*

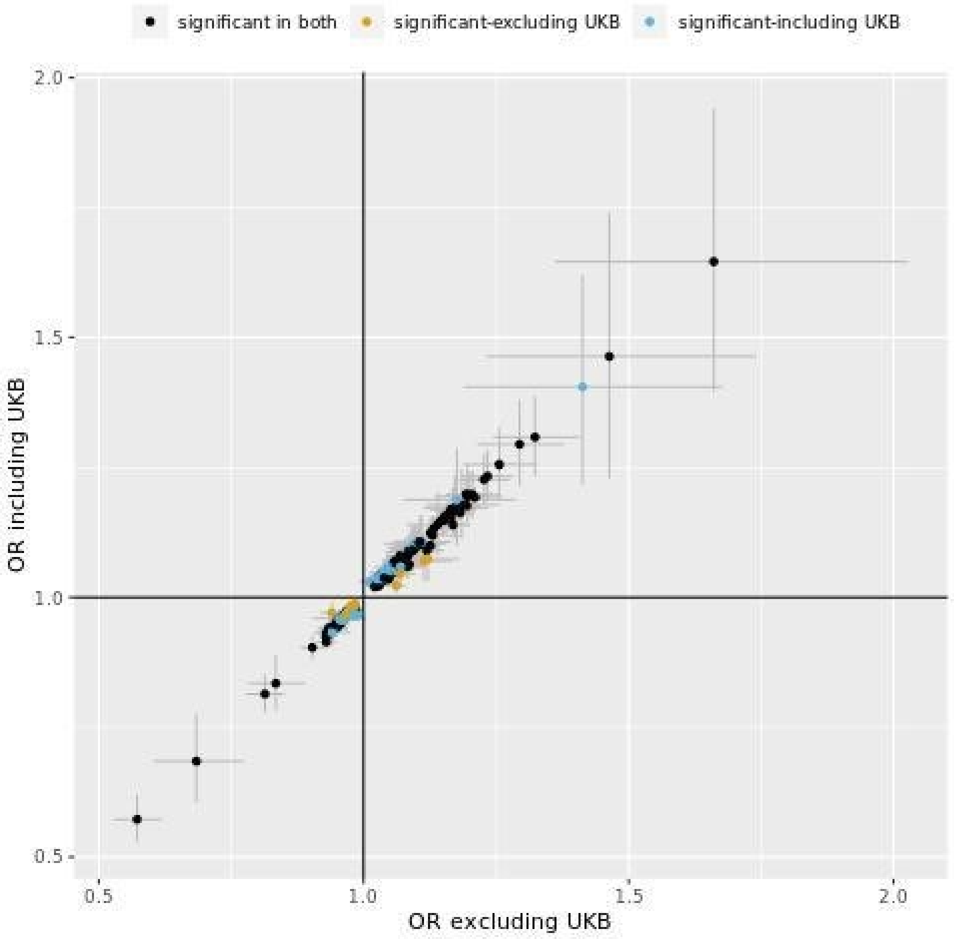


*Supplementary Figure 9: Comparison of the cross-ancestry sex-combined meta-analysis of Phenome Wide Association Studies (meta-PheWAS) including and excluding UK Biobank. The x-axis does include UK Biobank, whereas the y-axis includes UK Biobank (Supplementary Data 46). The blue dots denote significant hits including UK Biobank, the yellow dots denote significant hits excluding UK Biobank, and the black dots denote significant hits in both. The grey lines represent 95% confidence intervals.*

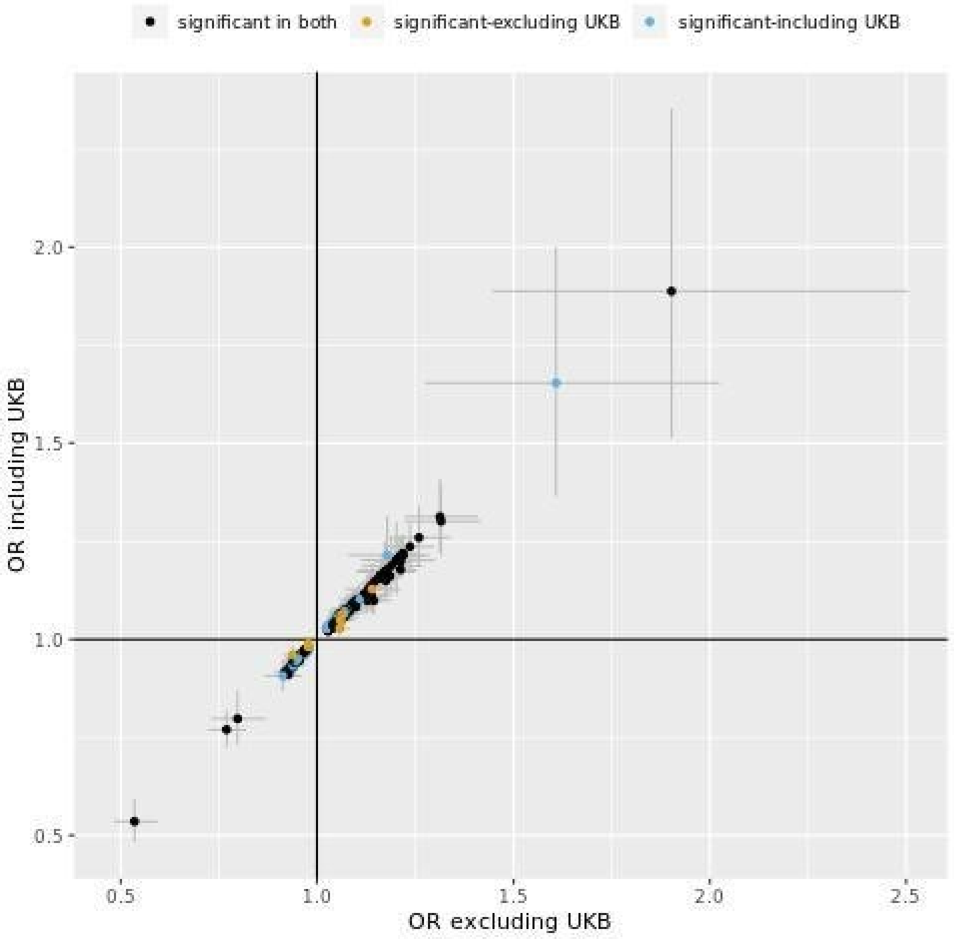


*Supplementary Figure 10: Comparison of the cross-ancestry male meta-analysis of Phenome Wide Association Studies (meta-PheWAS) including and excluding UK Biobank. The x-axis does include UK Biobank, whereas the y-axis includes UK Biobank (Supplementary Data 50). The blue dots denote significant hits including UK Biobank, the yellow dots denote significant hits excluding UK Biobank, and the black dots denote significant hits in both. The grey lines represent 95% confidence intervals.*

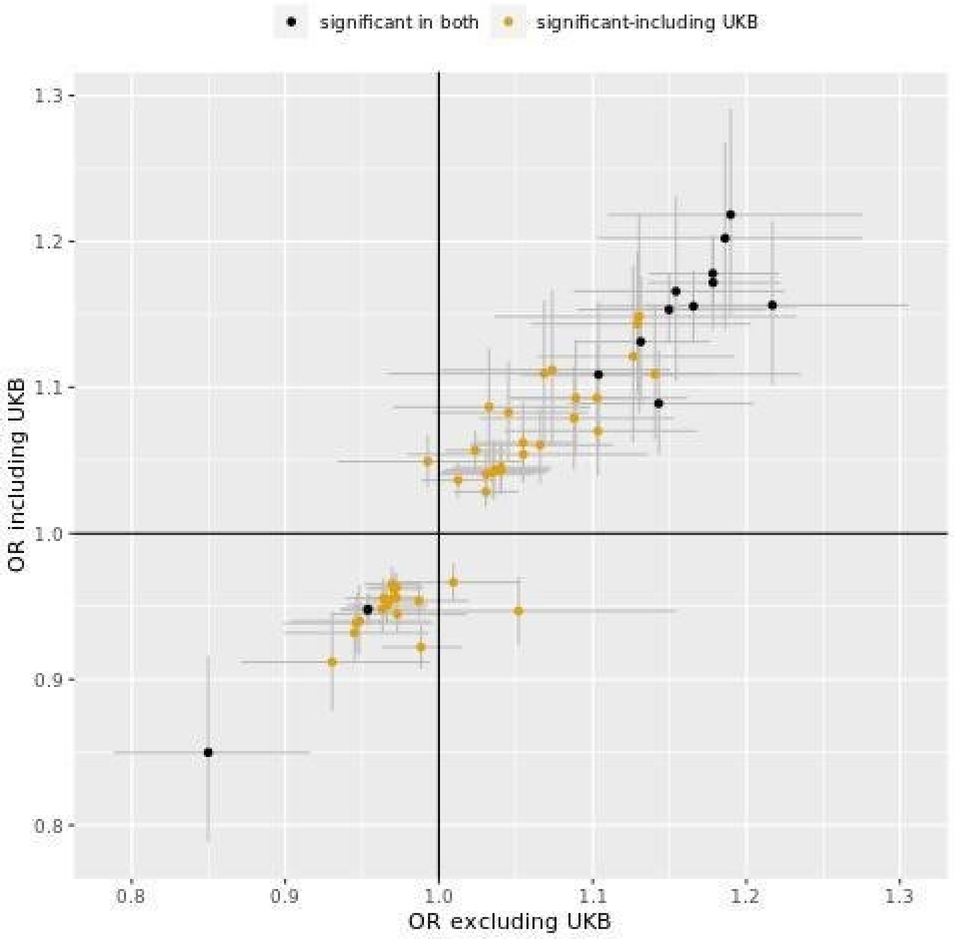


*Supplementary Figure 11: Comparison of odds ratio (OR) estimates between the cross-ancestry female meta-analysis of Phenome Wide Association Studies (meta-PheWAS) including and excluding UK Biobank. The x-axis does include UK Biobank, whereas the y-axis includes UK Biobank (Supplementary Data 54). The yellow dots denote significant hits including UK Biobank, and the black dots denote significant hits in both. The grey lines represent 95% confidence intervals.*

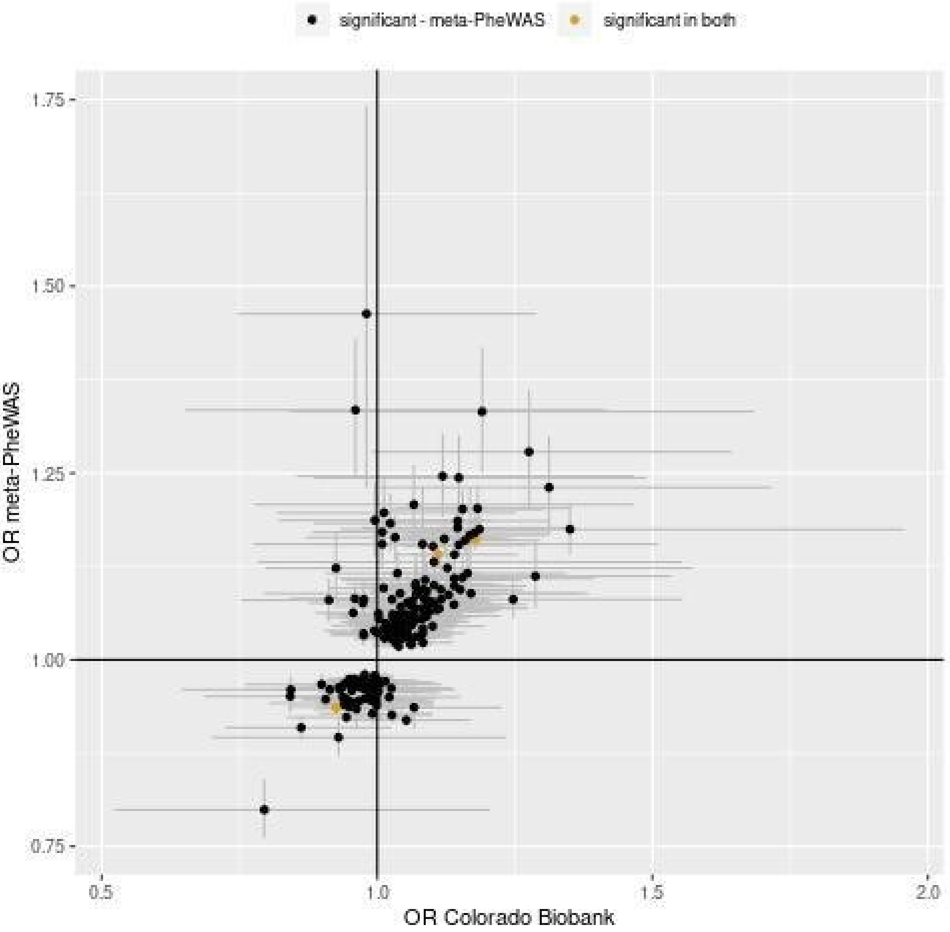


*Supplementary Figure 12: Comparison of odds ratio (OR) estimates between the sex-combined European ancestry meta-analysis of Phenome Wide Association Studies (meta-PheWAS) (y-axis) and the sex-combined European ancestry PheWAS in Colorado Biobank (x-axis), for the signals below Bonferroni threshold. The yellow dots are the significant ones for both, and the black dots are the significant hits for the sex-combined European ancestry meta-PheWAS. The grey lines represent 95% confidence intervals.*

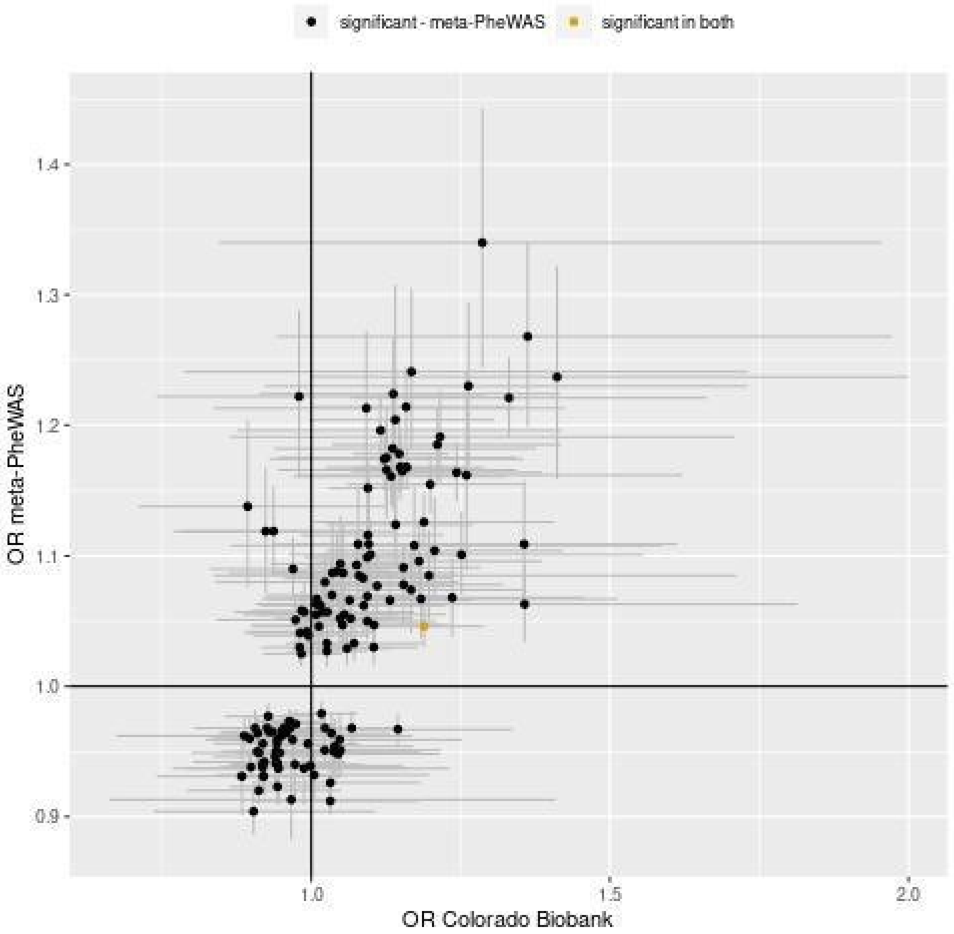


*Supplementary Figure 13: Comparison of odds ratio (OR) estimates between the male European ancestry meta-analysis of Phenome Wide Association Studies (meta-PheWAS) (y-axis) and the male European ancestry PheWAS in Colorado Biobank (x-axis), for the signals below Bonferroni threshold. The yellow dots are the significant ones for both, and the black dots are the significant hits for the male European ancestry meta-PheWAS. The grey lines represent 95% confidence intervals.*

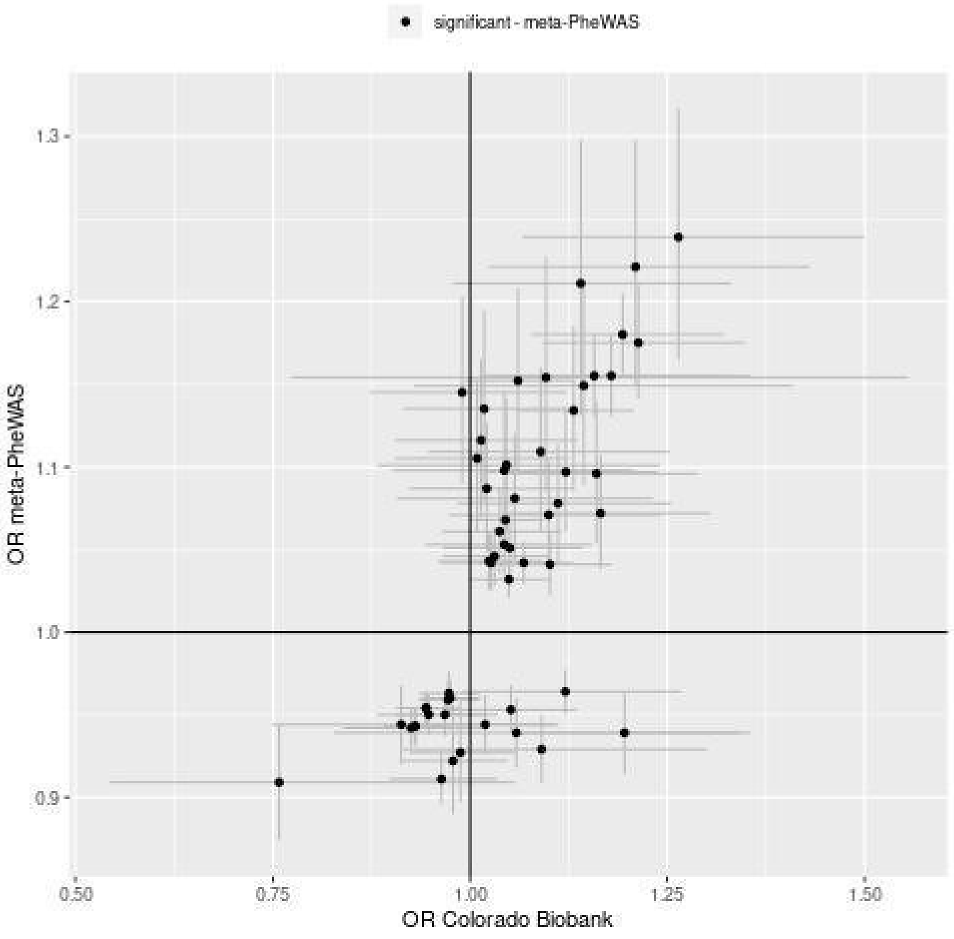


*Supplementary Figure 14: Comparison of odds ratio (OR) estimates between the female European ancestry meta-analysis of Phenome Wide Association Studies (meta-PheWAS) (y-axis) and the female European ancestry PheWAS in Colorado Biobank (x-axis), for the signals below Bonferroni threshold. The black dots are the significant ones for the female European ancestry meta-PheWAS. The grey lines represent 95% confidence intervals.*

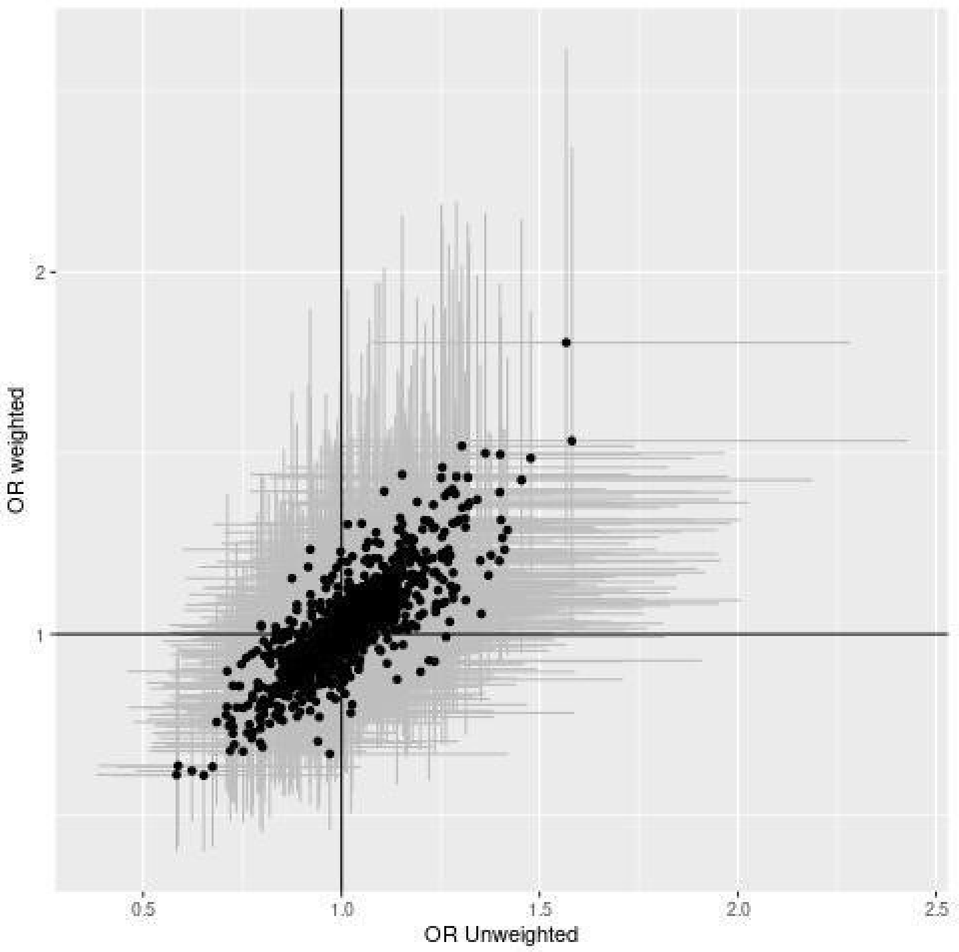


*Supplementary Figure 15: Comparison of odds ratio (OR) estimates between the sex-combined European ancestry weighted Phenome Wide Association Study (PheWAS) (y-axis) and unweighted PheWAS in Colorado Biobank (x-axis). The grey lines represent 95% confidence intervals.*

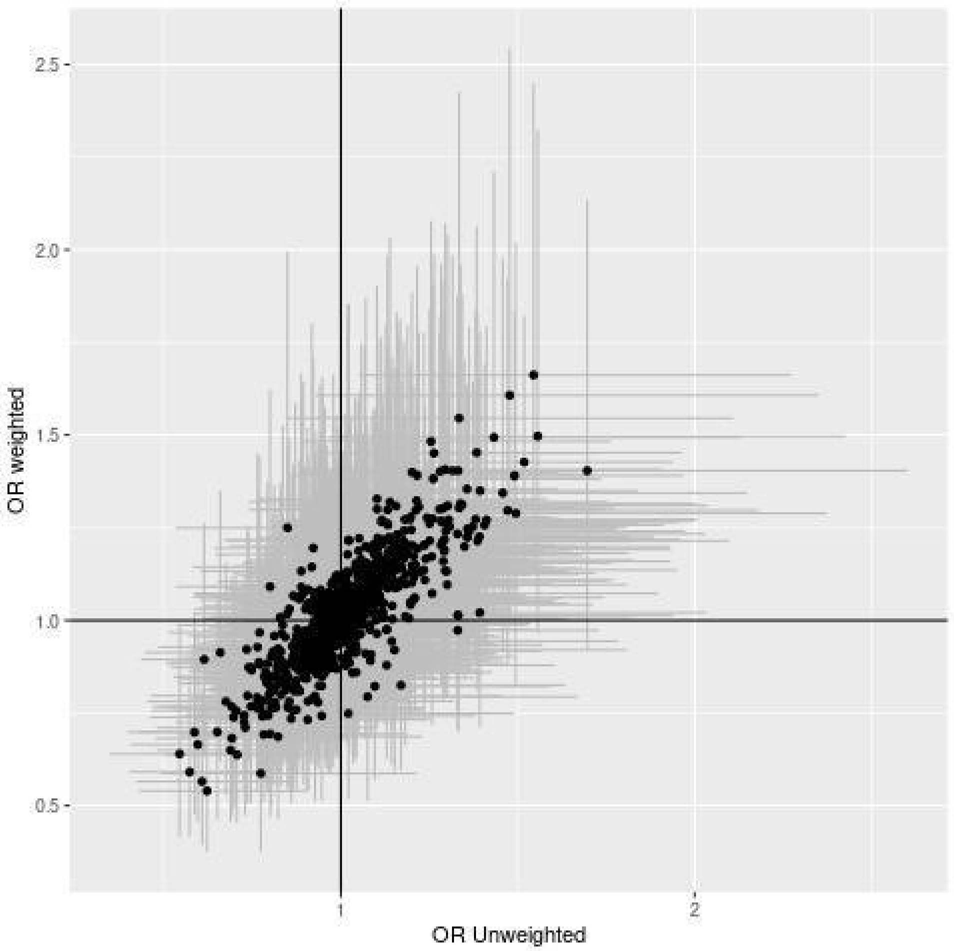


*Supplementary Figure 16: Comparison of odds ratio (OR) estimates between the male European ancestry weighted Phenome Wide Association Study (PheWAS) (y-axis) and unweighted PheWAS in Colorado Biobank (x-axis). The grey lines represent 95% confidence intervals.*

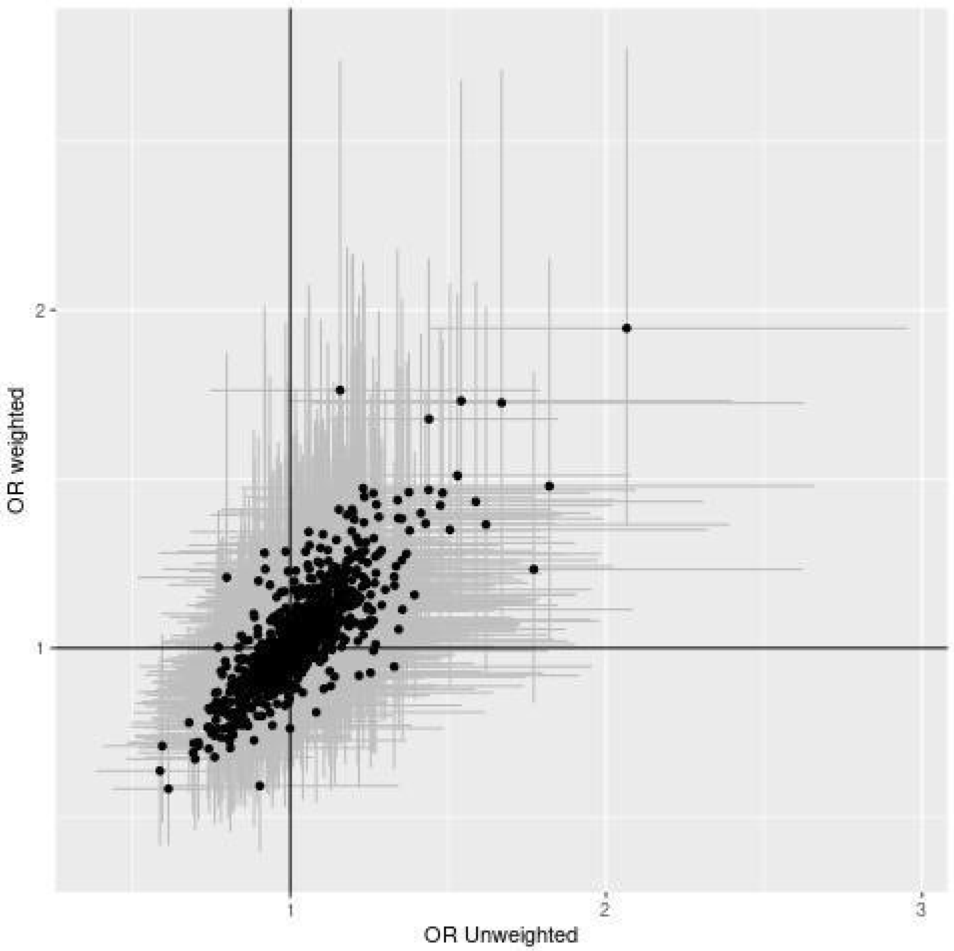


*Supplementary Figure 17: Comparison of odds ratio (OR) estimates between the female European ancestry weighted Phenome Wide Association Study (PheWAS) (y-axis) and unweighted PheWAS in Colorado Biobank (x-axis). The grey lines represent 95% confidence intervals.*
